# Effect of Maternal Probiotic and Piglet Dietary Tryptophan Level on Performance and Piglet Intestinal Health Parameters Pre-Weaning

**DOI:** 10.3390/microorganisms13061264

**Published:** 2025-05-29

**Authors:** Dillon P. Kiernan, John V. O’Doherty, Marion T. Ryan, Torres Sweeney

**Affiliations:** 1 School of Veterinary Medicine, University College Dublin, Belfield, D04 W6F6 Dublin, Ireland; dillon.kiernan@ucdconnect.ie (D.P.K.); marion.ryan@ucd.ie (M.T.R.); 2 School of Agriculture and Food Science, University College Dublin, Belfield, D04 W6F6 Dublin, Ireland; john.vodoherty@ucd.ie

**Keywords:** maternal transmission, microbiota, *Bacillus* spp., functional amino acid, aminobiotic, creep feed, gene expression, morphology, volatile fatty acids

## Abstract

A 2 × 3 factorial design was used to examine the effects of maternal probiotic supplementation (*Bacillus subtilis* and *Bacillus amyloliquefaciens*) and/or piglet dietary Trp levels on sow performance and fecal microbiota composition, as well as offspring pre-weaning performance and intestinal health parameters on the day of weaning. On day 83 of gestation, 48 sows were allocated to either: (1) control, or (2) control + probiotic (1.1 × 10^9^ colony forming units/kg of feed). Their litters were assigned to 0.22, 0.27, or 0.33% standardized ileal digestible (SID) Trp diets (0.17, 0.21 and 0.25 SID ratio of Trp to lysine (Trp:Lys), SID lysine = 1.3%). At weaning, one piglet per litter was sacrificed for intestinal health analysis. Diet had no effect on sow reproductive or offspring growth performance pre-weaning (*p* > 0.05). Maternal probiotic supplementation led to distinct microbial communities in the sow feces on day 114 of gestation, increasing the relative abundance of *Anaerocella* and *Sporobacter*, while decreasing *Lactobacillus*, *Ruminococcus*, and *Christensenella* (*p* < 0.05). In the offspring colonic digesta, maternal probiotic supplementation increased *Dorea*, *Sporobacter*, and *Anaerobacterium*, while reducing the potentially harmful phylum Proteobacteria, specifically the family Enterobacteriaceae (*p* < 0.05), with a tendency for a reduction in the genus *Escherichia* (*p* < 0.1). Maternal probiotic supplementation enhanced duodenal morphology and modulated the expression of genes in the ileum, including a downregulation of certain immune and barrier defense genes (*p* < 0.05). Piglets from probiotic sows had reduced branch chain fatty acids (BCFA) in the cecal digesta and an increase in the total VFA and acetate in the colonic digesta (*p* < 0.05). There were limited effects of Trp level in the offspring’s creep diet or maternal × creep interactions, though this analysis was likely confounded by the low creep feed intake (total of ~0.83 kg/litter).

## 1. Introduction

The microbiota of the gastrointestinal tract (GIT) plays fundamental roles in intestinal health and function, subsequently affecting many aspects of animal production, including feed efficiency [1], growth performance [2], and defense against pathogenic infections [3]. Birth and weaning represent two critical timepoints during which the GIT microbiota community undergoes assembly and transition, respectively. These vulnerable periods can foster environments that enable the opportunistic colonization and proliferation of enteric pathogens, with detrimental consequences for health and performance [4,5,6]. Antimicrobials and antibiotics, now banned due to escalating concerns about antimicrobial resistance, were traditionally utilized to reduce and treat incidences of dysbiosis. Through a process known as “priority effects”, microbial communities that colonize during early life can initiate a chain of microbial succession and influence the microbial populations that persist throughout life [7,8,9,10]. Furthermore, microbial exposure and colonization in early life has a fundamental role in the development of the GIT and the immune system [11,12,13,14]. However, consensus on what constitutes “advantageous” microbial exposure for optimal development remains elusive.

The composition of the GIT microbiota in postnatal pigs can be influenced by various microbial sources, with the sow being one of the primary contributors [15,16,17,18,19,20]. The postnatal period is a compelling window to promote offspring health and development, with manipulation of the maternal microbiota emerging as a promising strategy, as reviewed in detail by Kiernan et al. [21]. The addition of probiotics to the maternal diet can positively modulate the sow microbiota, elevating the proportions of beneficial microbes and their metabolites while decreasing the prevalence of potentially pathogenic microbes [22,23,24].

Positive alterations to the composition of the maternal microbiota can benefit the sow’s health and facilitate the transmission of beneficial microbes, including the supplemented probiotic strain, to the offspring [25,26]. *Bacillus* spp. are spore-forming bacteria that are noted for their stability and therefore particularly suited for use in animal feed-handling operations [27]. The combination of *Bacillus subtilis* and *Bacillus amyloliquefaciens* in maternal sow diets improves birth weights [28] and promotes offspring weight gain and weaning weights, both when supplemented solely to the sow [29] and when combined with direct piglet supplementation [28,30]. While individual supplementation of *Bacillus subtilis* or *Bacillus amyloliquefaciens* can modulate the sow’s fecal microbiota composition [31], the effects of their combined supplementation have only been analyzed in relation to specific bacterial counts [29]. The current study is a companion to our recent work, in which maternal *Bacillus subtilis* and *Bacillus amyloliquefaciens* supplementation enhanced offspring stomach function by upregulating genes involved in gastric acid secretion in the young pig [32].

In addition to its role in protein synthesis, tryptophan (Trp), an essential amino acid in pig diets, is involved in three major catabolic pathways that produce important bioactive metabolites. Of particular interest in this study are the immune modulatory effects of the metabolites produced via the kynurenine pathway, mediated by the host, and the indole pathway, mediated by the microbiota [33,34,35]. Via the stimulation of the aryl hydrocarbon receptor (AhR), Trp metabolites can promote immune homeostasis and immune tolerance [34,35]. Interestingly, increasing Trp supplementation prior to challenge can alleviate the negative effects on intestinal inflammation, barrier function, and oxidative stress in a post-weaned pig model [36,37,38]. However, ensuring the appropriate level of Trp is crucial, as too much Trp or over stimulation of the AhR can have negative effects on gut morphology and barrier function [39,40]. Furthermore, “aminobiotics” have recently been proposed as an innovative prebiotic group by Beaumont et al. [41], with Trp being recognized for its beneficial microbiota-modulating capabilities, as reviewed in [42] and investigated in post-weaned pigs [36,43,44,45]. Despite the potential beneficial effects on both the immune system and microbiota composition, the effect of pre-weaning creep Trp levels on the intestinal health and function at weaning has yet to be investigated in pigs.

Hence, the objectives of this study were twofold: firstly, to examine the effects of maternal probiotic (*Bacillus subtilis* and *Bacillus amyloliquefaciens*) supplementation on sow performance and fecal microbiota composition as well as offspring performance and intestinal health parameters pre-weaning; secondly, to evaluate the impact of increasing piglet dietary Trp levels, with or without maternal probiotic supplementation, on piglet performance and intestinal health parameters pre-weaning and to explore potential synergistic effects between maternal probiotic supplementation and increased piglet Trp levels.

## 2. Materials and Methods

The experimental procedures outlined in this study were approved by the University College Dublin Animal Research Ethics Committee (AREC-2022-ODoherty/AREC-2202-ODoherty) and were carried out in compliance with Irish law (SI no. 543/2012) and the EU Directive 2010/63/EU on animal research.

### 2.1. Experimental Design and Animal Management

The experimental design and animal management were as described in the companion study [32], with additional data and sample collection procedures as outlined below.

#### 2.1.1. Sow Management

A total of 48 crossbred sows (Large White × Landrace, Hermitage, Kilkenny, Ireland) were blocked by parity (mean parity 4.3 ± 2.5) and expected farrowing date (day 116 of gestation) and assigned to one of two dietary groups (*n* = 24 sows/diet): (1) basal diet (control) or (2) basal diet supplemented with a probiotic blend (*Bacillus subtilis* and *Bacillus amyloliquefaciens*). The probiotic supplement included *Bacillus subtilis* (DSM 25841) and *Bacillus amyloliquefaciens* (DSM 25840) and contained a minimum of 2.75 × 10^9^ colony-forming units (CFU) per gram, according to manufacture. Specifically, the product provided 1.50 × 10^9^ CFU per gram of *Bacillus subtilis* and 1.25 × 10^9^ CFU per gram of *Bacillus amyloliquefaciens*. The probiotic product (SOLPREME^®^, Chr. Hansen A/S, Hørsholm, Denmark) was provided by Chr. Hansen A/S. The probiotic was top-dressed on the feed to achieve a supplementation rate equivalent to 400 g of probiotic supplement per ton of gestation/lactation feed consumed, providing approximately 1.1 × 10^9^ CFU per kg of feed, as per manufacturer recommendations. The first feeding of the day, during both gestation and lactation, were top-dressed with the probiotic to ensure consumption. The parity distribution of sows was as follows: 20% were in their first parity, 40% were between their second and fourth parities, and 40% were in their fifth parity or beyond. The diets were fed from day 83 (±1.8 days) of gestation until the day of weaning, at day 26 (±1.8 days) of lactation. The ingredients and chemical composition analysis of the lactation and gestation diets was described and presented in [32] and shown in Table 1 and Table 2. The diets were formulated to meet or exceed National Research Council recommendations (NRC 2012).

From days 83 to 110 of gestation, the sows were kept in groups of six based on their dietary group. The temperature in the gestation room was maintained at 20 °C throughout the experiment. During this period, the sows received 3.1 kg/day of gestation feed. In the gestation room, the sows were fed in a shared trough (six sows per trough) with equal meals provided at 8 a.m. and 2 p.m.

On day 110 of gestation, the sows were relocated to individual farrowing pens (2.4 m × 1.8 m) equipped with crates, slatted floors, and heat pads for piglets. From day 110 to 113 of gestation, the sows received 2.9 kg/day of lactation feed. From day 113 until farrowing, the sows received 2.3 kg/day of lactation feed, and then, the feed supply was increased by 0.7 kg/day until day 3 postpartum. Following this, the sows were fed semi-ad libitum with the standard lactation diet, adjusted for each sow based on its daily intake. In the farrowing room, the sows were fed from individual troughs and provided three equal meals at 6 a.m., 11 a.m., and 3 p.m. The temperature in the farrowing room was maintained at approximately 24 °C during farrowing and gradually reduced to 20 °C by day 10 post farrowing. The sows had ad libitum access to fresh drinking water throughout the experimental period, which was provided through nipple drinkers.

#### 2.1.2. Piglet Management

**Figure 1:**
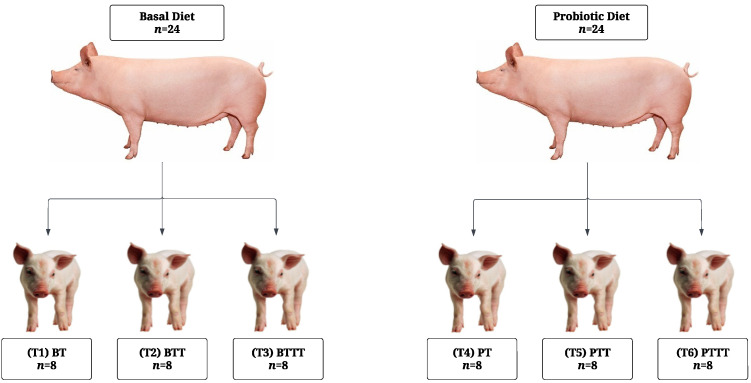
Experimental design overview.

All farrowings were supervised. Every piglet in each litter was individually weighed and tagged at birth. Four piglets (two male and two female) near the median birth weight were selected per sow and excluded from cross-fostering. Cross-fostering occurred between 12 and 24 h postpartum within maternal dietary groups to equalize litter size (*n* = 14). All piglets received an intramuscular injection of iron (Uniferon, Pharmacosmos A/S, Holbæk, Denmark) on day 1 postpartum. On day 8 postpartum, both maternal dietary groups were sub-blocked into three groups based on parity (mean parity 4.3 ± 2.5), litter age (7 ± 1.8 days), and litter size (13.25 ± 0.9 pigs). Litters were then assigned to one of three creep diets: 0.22, 0.27 or 0.33% SID Trp, corresponding to 0.17, 0.21, and 0.25 SID Trp/Lys. The ingredient composition and analysis of the creep diets are presented in Table 1 and Table 2. Trp was supplemented in the diets in the form of L-Trp (>98% purity, Ajinomoto Health and Nutrition, Paris, France). The two factors, maternal diet and creep diet, were arranged in a 2 × 3 factorial design, resulting in the following six experimental groups: (T1) BT (basal sows and piglets supplemented with 0.22% SID Trp); (T2) BTT (basal sows and piglets supplemented with 0.27% SID Trp); (T3) BTTT (basal sows and piglets supplemented with 0.33% SID Trp); (T4) PT (probiotic sows and piglets supplemented with 0.22% SID Trp); (T5) PTT (probiotic sows and piglets supplemented with 0.27% SID Trp); (T6) PTTT (probiotic sows and piglets supplemented with 0.33% SID Trp) (Figure 1).

### 2.2. Data and Sample Collection

#### 2.2.1. Data and Sample Collection—Sow

##### Sow Backfat and Feed Intake

Sow backfat was recorded using a digital backfat meter (Renco LEAN-MEATER, Renco Corporation) on day 114 of gestation and day 26 of lactation (weaning day). The meter probe was placed on the back of the sow at the level of the second last rib, approximately 6 cm from the side of the backbone. A reading was taken from both the right and left side of the sow’s back. The average of both readings was recorded, and lactation backfat loss was then calculated. Total lactation feed intake was recorded for each sow, and average daily feed intake was then calculated.

##### Sow Fecal Sampling

Fresh fecal samples (~20 g) were collected from each sow on day 114 of gestation. The samples were collected in sterile containers (Sarstedt, Wexford, Ireland) and immediately snap-frozen on dry ice before being stored at −80 °C.

#### 2.2.2. Data and Sample Collection—Piglet

##### Performance and Creep Intake

Individual piglet body weights were recorded at birth and days 7, 21, and 26 postpartum (weaning), and the daily gain was calculated. Creep feed intake was recorded daily on a litter basis and summed at the point of weaning to calculate total litter creep intake.

##### Piglet Tissue and Digesta Sampling

At weaning (day 26), one piglet per litter (one of four selected at birth) (*n* = 8/dietary group) (8.4 kg bodyweight) was humanely sacrificed with a lethal injection with pentobarbitone sodium (Euthatal Solution, 200 mg/mL; Merial Animal Health) at a rate of 0.71 mL/kg body weight to the cranial vena cava. Euthanasia was completed by a competent person in a room separate from other piglets. When selecting the pig to be sacrificed, from the four pigs/litter selected at birth, the heaviest pigs were preferred to standardize the selection process. The piglets were not fasted prior to sacrifice. The entire gastrointestinal tract was immediately removed. The pH of the stomach was measured at the center of the lumen using a pH probe meter (HI-98190, Hanna Instruments, Padovana, Padua, Italy). Tissue samples from the duodenum and ileum were collected for gene expression analysis. The tissue sections were removed, dissected along the mesentery, rinsed in PBS, and then stripped of the overlying smooth muscle. Sections of tissue (1 cm^2^) were then stored in RNAlater^®^ solution (5 mL) overnight at 4 °C. The RNAlater^®^ was removed 24 h later and the samples stored at −80 °C. For gut morphological analysis, a section from the duodenum (located 10 cm distal from the stomach) was excised and fixed in 10% neutral-buffered formalin. Cecal and colonic digesta was aseptically collected in sterile containers (Sarstedt, Wexford, Ireland), snap-frozen on dry ice, and stored at −80 °C for subsequent VFA analysis and 16S rRNA sequencing of the colonic digesta. For the QPCR quantification of selected bacterial populations in the ileal mucosa-associated microbiota, the ileum was longitudinally incised, the digesta was gently removed, and the mucosal layer was separated from the underlying muscle using a glass slide, placed in sterile containers, snap-frozen on dry ice, and stored at −80 °C until subsequent QPCR analysis.

### 2.3. Analysis

#### 2.3.1. Microbial Analysis

16S rRNA sequencing analysis was conducted on sow fecal samples collected on day 114 of gestation and offspring colonic digesta collected at weaning. QPCR analysis was conducted on ileal mucosal samples collected at weaning for the absolute quantification of *Escherichia coli*. For the offspring samples, only probiotic vs. control diets were analyzed. Creep diet was excluded from the analysis given the low creep feed intakes achieved in the study.

##### Microbial DNA Extraction

Microbial genomic DNA was isolated from sow fecal and offspring colonic digesta and ileal mucosal scraping samples utilizing the QIAamp PowerFaecal Pro DNA stool kit (Qiagen, West Sussex, UK) in accordance with the manufacturer’s guidelines. The Nanodrop ND-1000 Spectrophotometer (Thermo Scientific, Wilmington, DE, USA) was used to evaluate the quantity and quality of the extracted DNA.

##### Illumina Sequencing and Bioinformatic Analysis of Sow Feces and Offspring Colonic Digesta

Sequencing of the V3–V5 hypervariable region of the bacterial 16S rRNA gene was conducted on an Illumina MiSeq platform following standard protocols (Eurofins Genomics, Ebersberg, Germany). In summary, the V3–V5 region underwent PCR amplification with universal primers containing adapter overhang nucleotide sequences for forward and reverse index primers. Subsequently, AMPure XP beads (Beckman Coulter, Indianapolis, IN, USA) were utilized for amplicon purification, followed by an index PCR using Nextera XT index primers (Illumina, San Diego, CA, USA). The indexed samples were purified with AMPure XP beads, quantified using a fragment analyzer (Agilent, Santa Clara, CA, USA), and combined in equal quantities. The resulting pooled library was quantified using the Bioanalyzer 7500 DNA kit (Agilent, Santa Clara, CA, USA) and subjected to sequencing employing v3 chemistry (2 × 300 bp paired end reads). The bioinformatic analysis of the resulting sequences was performed by Eurofins Genomics (Ebersberg, Germany) using the open-source software package (version 1.9.1) Quantitative Insights into Microbial Ecology (QIIME) [47]. All raw reads passing the standard Illumina chastity filter were demultiplexed according to their index sequences (read quality score > 30). The primer sequences were clipped from the start of the raw forward and reverse reads. Where primer sequences were not perfectly matched, read pairs were removed to retain only high-quality reads. Paired-end reads were merged, if possible, to obtain a single, longer read that covered the full target region using the software FLASH 2.2.00 [48]. Pairs were merged with a minimum overlap size of 10 bp to reduce false-positive merges. The forward read was only retained for the subsequent analysis steps when merging was not possible. Merged reads were quality-filtered according to the expected length and known length variations of the V3–V5 region (ca. 535 bp). The ends of retained forward reads were clipped to a total read length of 283 bp to remove low-quality bases. Merged and retained reads containing ambiguous bases were discarded. The filtered reads (merged and quality clipped retained forward reads) were used for the microbiome profiling. Chimeric reads were identified and removed based on the de novo algorithm of UCHIME [49] as implemented in the VSEARCH package [50]. The remaining set of high-quality reads were processed using minimum entropy decomposition (MED) to partition reads to operational taxonomic units (OTU) [51,52]. DC-MEGABLAST alignments of cluster representative sequences to the NCBI nucleotide sequence database were performed for taxonomic assignment (from phylum to genus) of each OTU. A sequence identity of 70% across at least 80% of the representative sequence was the minimal requirement for considering reference sequences. Abundances of bacterial taxonomic units were normalized using lineage-specific copy numbers of the relevant marker genes to improve estimates [53]. The data were then analyzed as previously described in [54]. Briefly, the data matrix was constructed using the normalized OTU table, phenotype metadata, and phylogenetic tree. This matrix was subsequently imported into the phyloseq package in R (Version 3.5.0). Richness and diversity dynamics within the microbiota were evaluated using indices such as observed richness, Chao1, Shannon, Simpson, inverse Simpson, and Fisher. Beta diversity measurements quantified differences in the phylogenetic structure of OTUs between a given sample and all others, following normalization to ensure comparability of taxonomic feature counts across samples. To calculate the distance matrix for multidimensional reduction processes, the non-phylogenetic Bray–Curtis distance metric was employed using the phyloseq package. Differential abundance analyses were conducted on datasets extracted from the phyloseq object at the phylum, family, and genus levels.

##### Absolute Quantification of *Escherichia coli* in the Offspring Ileal Mucosa

Previously validated primers for *Escherichia coli* (Table 3) were used in all QPCR reactions (DNA extractions and standard DNA). To construct the standard, which would serve as a template for the validated primer sets, sequences that included the forward and the reverse complement of the reverse primer for bacterial groups of interest were arranged in a staggered manner ensuring that each primer pair generated a PCR product 115 bp, an optimal size range for QPCR. Synthesis of the DNA construct, incorporation into pUC-GW and a miniprep was performed by Genewiz-Azenta (Leipzig, Germany). The vector (1 ug), containing the insert, was linearized using the restriction enzyme AscI, Part No. 10715651 (Thermo Fisher Scientific, Waltham, MA, USA), according to the manufacturer’s instructions, and purified using GenElute^TM^ PCR Clean-Up Kit (Sigma-Aldrich, St. Louis, MO, USA). The purified linearized plasmid was quantified on a Nanodrop Spectrophotometer (Thermo Scientific), and the concentration was converted to copy number using the following formula. DNA copies/µL = DNA concentration, in ng/µL* 6.022 × 1023/Length of the template in bp. Serial dilutions (10-fold) of the linearized vector were performed in TE buffer (Part No. 12090015) (Thermo Fisher Scientific) with Lambda DNA, Part No. SD0011 (Thermo Fisher Scientific) added to a final concentration of (5 ng/µL). Dilutions (10-fold) ranged from 80 × 10^7^ to 80 × 10^−1^ copies/μL. This was used to generate a standard curve for the *Escherichia coli* assay. Dissociation curves were also performed to check for a single product and that there was no primer-dimer or non-specific binding. Finally, 3 μL of a 10-fold dilution of the extracted DNA was run in duplicate alongside the standard curve. For the QPCR, the final reaction volume (20 μL) included 1.5 μL template DNA, 1 μL of forward primer (10 μM), 1 μL of reverse primer (10 μM), 5 μL nuclease-free water, and 10 μL of GoTaq^®^ QPCR Master Mix (Promega, Madison, WI, USA) for the remaining bacterial groups. QPCR reactions were performed in duplicate on the ABI 7500 Fast PCR System (Applied Biosystems, Foster City, CA, USA) using the following cycling conditions: a denaturation step 95 °C for 10 min and 40 cycles of 95 °C for 15 s and 60 °C for 1 min. Bacterial counts were determined using the standard curve derived from the mean Ct value and the log-transformed gene copy number of the respective plasmid and expressed as log-transformed gene copy number/µL extracted DNA. To determine the gene copy number/ng, extracted DNA = (gene copy number/μL extracted DNA)/(DNA concentration ng/µL).

#### 2.3.2. Volatile Fatty Acid Analysis

Volatile fatty acid analysis was performed on the piglet cecal and colonic digesta. Gas liquid chromatography was used to determine the VFA concentrations, as described in detail in [55]. First, 1 g of digesta was diluted with water (2.5 × sample weight) and centrifuged (1400× *g* for 10 min) using a Sorvall GLC-2B centrifuge (DuPont, Wilmington, DE, USA). Then, 1 mL of supernatant and 1 mL of internal standard (0.05% 3-methyl-n-valeric acid in 0.15 mol/L oxalic acid dihydrate) were mixed with 3 mL of distilled water and then centrifuged for 10 min at (500× *g*). The supernatant was filtered with a syringe filter (0.45 polytetrafluoroethylene (TFE)) into a chromatographic vial. Approximately 1 µL was injected into a Varian 3800 GC (Ontario, Canada) with an ECTM 1000 Grace column (15 m × 0.53 mm I.D) with a film thickness of 1.20 µm. The temperature program was set to 75–95 °C, which increased by 3 °C/min, and 95–200 °C, which increased by 20 °C/min, and this was held for 0.5 min. The detector temperature was 280 °C, and the injector temperature was 240 °C.

#### 2.3.3. Gene Expression Analysis

Gene expression analysis was performed on piglet duodenal and ileal tissue. RNA extraction, cDNA synthesis, and QPCR were conducted as described in our companion study [32]. Briefly, RNA was extracted using TriReagent (Sigma-Aldrich, St. Louis, MO, USA), purified with the E.Z.N.A. ^®^ RNA kit (Omega Bio-Tek, Norcross, GA, USA), and reverse-transcribed using the High-Capacity cDNA Reverse Transcription Kit (Applied Biosystems, Foster City, CA, USA). Quantitative PCR was performed using 10 µL FastStart Universal SYBR Green Master Mix (Roche Diagnostics, Mannheim, Germany)), 1.2 µL primer mix (5 pM/µL), and 5 µL cDNA. Targets were selected based on previous work [56], and additional targets were included following reviews of the literature. The selected genes covered a range of gut health parameters, including immune response (*AHR*, *DEFB1*, *DEFB3*, *IL6*, *CXCL8*, *IL10*, *IL22*, *TLR2*, *TLR4*, and *TNF*), intestinal barrier function (*CLDN3* and *TJP1*), mucin production (*MUC1* and *MUC2*), nutrient transport (*FABP2*, *SLC2A1*, *SLC2A2*, *SLC15A1*, *SLC7A5*, *SLC7A6*, and *SLC7A7*), and oxidative status (*SOD2* and *NOX1*). Furthermore, Trp is a precursor for serotonin [57], which can be associated with the intestinal health in piglets [58,59]; hence, the serotonin receptor *HTR4* was also analyzed. The geometric mean of the reference genes *HMBS* and *YWHAZ* was used to normalize expression in the duodenum, while reference genes *H375A* and *ACTB* were used for the ileum. Reference gene pairs were selected based on their M value calculated by the GeNorm algorithm. Normalized relative quantities were calculated using qbase PLUS software (BioGazelle, Ghent, Belgium). Accession numbers, primer sequences, and amplicon lengths can be found in Table 4.

#### 2.3.4. Morphological Analysis

Morphological analysis was performed on the duodenal samples. Standard paraffin embedding techniques were used to prepare the tissue. A light microscope with an image analyzer (Image-Pro Plus, Media Cybernetics, Oxfordshire UK) was used to measure the villus height (VH), crypt depth (CD), and VH-to-CD ratio (VH/CD) at a magnification of 10×. Fifteen measurements of villi and crypt were taken for each section. The VH was measured from the crypt–villus junction to the tip of the villus, and CD was measured from the crypt–villus junction to the base. Results are expressed as mean VH or CD in μm.

#### 2.3.5. Statistical Analysis

Performance, morphology, gene expression, VFA, and *Escherichia coli* count data were analyzed as a 2 × 3 factorial. The model included the effects of maternal diet (control or probiotic) and creep diet (0.22, 0.27, or 0.33% Trp) and their associated two-way interactions. The data were initially checked for normality using the univariate procedure on Statistical Analysis Software (SAS) (v 9.4) (SAS Institute, Cary, NC, USA) and transformed if necessary. The performance, stomach pH, gene expression, morphology, bacterial gene copy number, and VFA data were then analyzed using the general linear model (PROC GLM) procedure of SAS. Gene expression *p*-values were Bonferroni-adjusted. The sow fecal and offspring colonic digesta microbiome data were analyzed using the generalized linear mixed model (PROC GLIMMIX) procedure of SAS, with *p*-values Benjamini–Hochberg-adjusted. The results are presented as least square means with their standard errors. The probability level that denoted significance was *p* < 0.05. Within R (Version 3.5.0), the rcorr function from the Hmisc package [60] was used to compute the correlation matrix and *p*-values. The ggplot2 package [61] within R was then used to visualize the correlation matrix, while reshape2 was used to format the data for visualization.

## 3. Results

### 3.1. Sow Reproductive and Offspring Performance

The effects of maternal probiotic supplementation and piglet dietary Trp level on sow and offspring performance is presented in Table 5.

There was no maternal or creep effect on piglet mortality, piglet weight, litter weight, or daily gain at any timepoint, nor was there an effect on litter creep intake, sow feed intake, or sow backfat loss (*p* > 0.05). There was a maternal × creep interaction on litter size at day 21 (*p* < 0.05), with litter size being greater from probiotic sows litters that received 0.33% Trp compared to probiotic sow litters that received 0.22% Trp (*p* < 0.05), an effect not present in litters from control sows (*p* < 0.05).

### 3.2. Stomach pH at Weaning

Stomach pH was similar between offspring from probiotic sows compared to offspring from control sows (3.46 vs. 3.68, SEM = 0.19) and piglets who received 0.22%, 0.27%, or 0.33% Trp (3.46 vs. 3.67 vs. 3.59, SEM = 0.23) (*p* > 0.05). There was no maternal × creep interaction on stomach pH (*p* > 0.05).

### 3.3. Duodenal Morphology

The effects of maternal probiotic supplementation and piglet dietary Trp level on duodenal morphology are presented in Table 6. Offspring from probiotic sows had increased VH and VH/CD ratio and reduced CD compared to offspring from control sows (*p* < 0.05). There was no effect of creep or maternal × creep interaction on VH, CD, or VH:CD (*p* > 0.05).

### 3.4. Small Intestine Gene Expression

The effects of maternal probiotic supplementation and piglet dietary Trp level on the relative expression of immune, barrier defense, oxidative status, and nutrient transporter genes in the duodenum and ileum are presented in Table 7 and Table 8, respectively.

#### 3.4.1. Duodenum

Offspring from probiotic sows had upregulated *SOD2* expression compared to offspring from control sows (*p* < 0.05). There was a maternal × creep interaction on the expression of *IL10* (*p* < 0.05); offspring from probiotic sows fed 0.33% Trp had upregulated *IL10* compared to offspring from probiotic sows fed 0.27% Trp (*p* < 0.05), but this effect was not observed in offspring from control sows (*p* > 0.05).

#### 3.4.2. Ileum

In the ileum, there were significant changes to the expression of genes in offspring from probiotic sows compared to offspring from control sows, with no effects of creep or maternal × creep interactions. Offspring from probiotic sows had downregulated *DEFB1*, *TLR2*, *CLDN3*, *MUC1*, *SLC7A7*, *SOD2*, and *HTR4* and upregulated *SLC7A5* compared to offspring from control sows (*p* < 0.05).

#### 3.4.3. Correlation of Duodenal Gene Expression and Physiological Parameters

A correlation analysis of the duodenal morphology and gene expression data, in combination with body weight, age at weaning, creep intake, and stomach pH was performed. For presentation purposes, the results were organized into two main categories: (1) nutrient transporter gene expression and (2) immune response and barrier defense gene expression.

##### Nutrient Transporters

**Figure 2:**
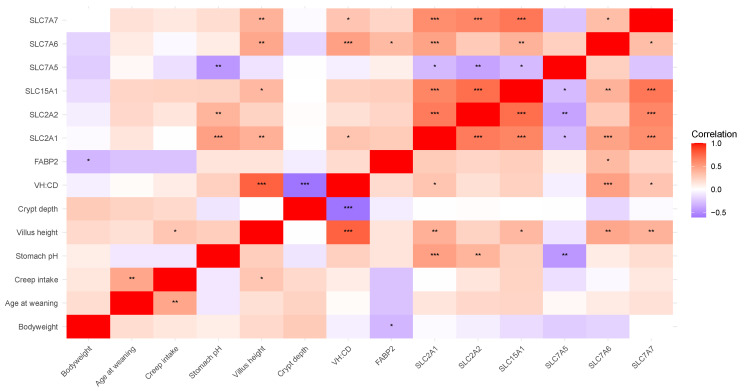
Correlation matrix illustrating Pearson correlations of duodenal nutrient transporter gene expression and physiological parameters. Positive (red) and negative (purple) correlations are represented in color strength on a scale of −1 to 1. * *p* < 0.05; ** *p* < 0.01; *** *p* < 0.001. VH/CD, villus height-to-crypt depth ratio.

The correlations of duodenal nutrient transporter expression and physiological parameters are presented in Figure 2. As expected, there was a positive correlation between VH and VH/CD as well as a positive correlation between creep intake and age at weaning and a negative correlation between VH/CD and CD. Body weight correlated negatively with *FABP2* expression. Aside from the positive correlation with creep intake, age at weaning had no other correlations. Stomach pH was positively correlated to *SLC2A1* and *SLC2A2* and negatively correlated to *SLC7A5* expression. There was a positive correlation between VH and creep intake and between VH and the expression of several nutrient transporter genes (*SLC7A7*, *SLC7A6*, *SLC15A1*, and *SLC2A1*). The analyzed nutrient transporters exhibited positive correlations with each other except for *SLC7A5*, which was negatively correlated with the expression of *SLC2A1*, *SLC2A2*, and *SLC15A1*. Additionally, *FABP2* was correlated to *SLC5A6* but not to the expression of any of the other nutrient transporters analyze.

##### Immune Response and Barrier Defense

**Figure 3:**
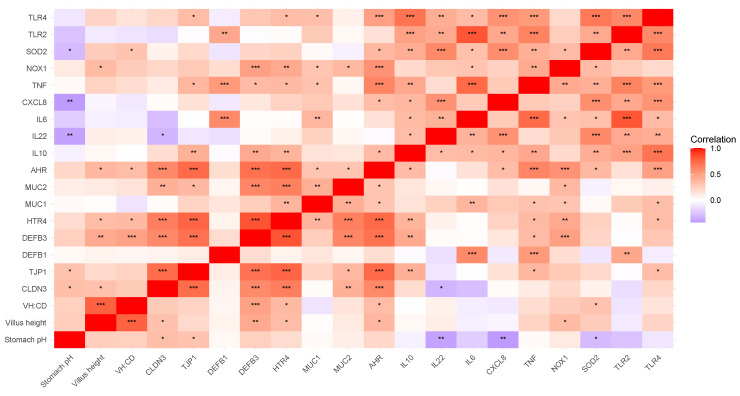
Correlation matrix illustrating Pearson correlations of duodenal immune and barrier defense gene expression and physiological parameters. Positive (red) and negative (purple) correlations are represented in color strength on a scale of −1 to 1. * *p* < 0.05; ** *p* < 0.01; *** *p* < 0.001. For presentation purposes, creep intake, age at weaning, and crypt depth were removed, as they had no correlations in the current plot; body weight was removed, as it was only correlated to *TJP1* expression. VH/CD, villus height-to-crypt depth ratio.

The correlations between duodenal immune response, barrier defense, and serotonin receptor expression and physiological parameters are presented in Figure 3. Both VH and VH/CD were positively correlated with *DEFB3*, *HTR4*, and *AHR* expression. Additionally, VH was positively correlated with *CLDN3* and *NOX1*, while VH/CD was positively correlated with *SOD2*. Surprisingly, stomach pH was positively correlated with tight-junction proteins *TJP1* and *CLDN3* but negatively correlated with the expression of *CXCL8*, *IL22*, and *SOD2*. There were several notable correlations among genes. Specifically, the beta-defensin genes *DEFB1* and *DEFB3* exhibited distinct co-expression patterns. *DEFB1* was positively correlated with *TLR2*, *IL6*, and *TNF* expression, whereas *DEFB3* was positively correlated with VH, VH/CD, tight junction proteins, *TJP1* and *CLDN3*, *HTR4*, and *MUC2*, among others. In addition to their positive correlations with VH and VH/CD, *AHR* and *HTR4* were positively correlated to each other and several immune-related genes: *CLDN3*, *TJP1*, *DEFB3*, *MUC1*, and *MUC2*. Additionally, *AHR* was positively correlated to *CXCL8*, *SOD2*, and *TLR4* expression.

### 3.5. Volatile Fatty Acids

The effects of maternal probiotic supplementation and piglet dietary Trp level on cecal and colonic digesta VFA concentrations in the offspring at weaning are presented in Table 9.

#### 3.5.1. Cecal Volatile Fatty Acids

In the cecal digesta, offspring from probiotic sows had reduced iso-butyrate, iso-valerate, valerate, and BCFA per mmol/g digesta (*p* < 0.05). There were no creep or maternal × creep interactions on VFA concentrations in the cecal digesta (*p* > 0.05).

#### 3.5.2. Colonic Volatile Fatty Acids

In the colonic digesta, offspring from probiotic sows had an increased total VFA per mmol/g digesta (*p* < 0.05). Additionally, offspring from probiotic sows had an increased concentration of acetate in the colonic digesta (*p* < 0.05). Piglets fed 0.22% Trp had an increased valerate concentration in the colonic digesta compared to piglets fed 0.27% Trp and 0.33% Trp (*p* < 0.05). There was no maternal × creep interaction on VFA concentrations in the colonic digesta (*p* > 0.05).

### 3.6. Microbial Composition

#### 3.6.1. 16S rRNA Microbial Analysis of Sow Feces

##### Bacterial Richness and Diversity of Sow Feces

**Figure 4:**
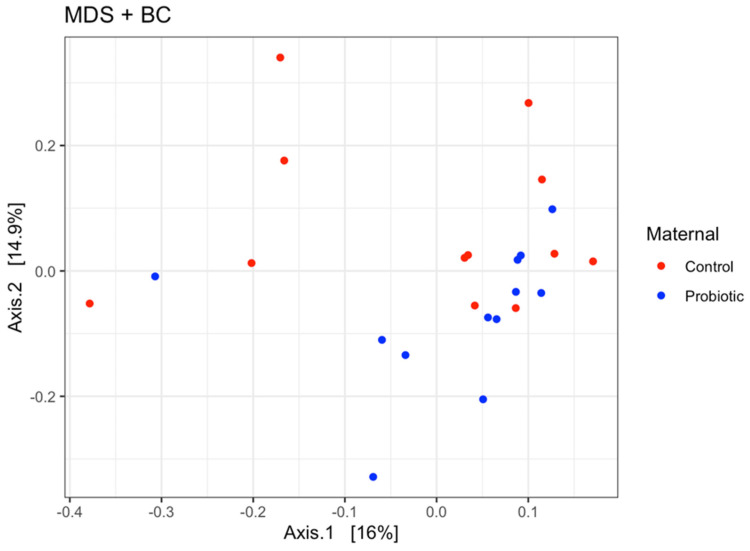
Beta diversity of sow feces on day 114 of gestation as grouped by maternal diet and based on the Bray–Curtis distance matrix and visualized using multi-dimensional scaling.

The effects of probiotic supplementation on the measures of beta diversity and alpha diversity in the sow feces on day 114 of gestation are presented in Figure 4 and Table 10, respectively. There was a difference in beta diversity between the control and probiotic sow feces based on a PERMANOVA analysis of Bray–Curtis dissimilarity (*p* < 0.05). However, probiotic supplementation had no effect on the Observed, Chao1, Shannon, Simpson, InvSimpson, or Fisher index measures of alpha diversity in the sow feces (*p*  > 0.05).

##### Differential Microbial Abundance Analysis of Sow Feces

All data on differential bacterial abundances at phylum, family, and genus level in the sow feces are presented in Table 11.

- Phylum Level—Sow Feces

At phylum level, six bacterial phyla were identified in total across all sow fecal samples: Firmicutes (65.1%), Bacteroidetes (30.2%), Tenericutes (1.3%), Actinobacteria (0.8%), Spirochaetes (0.4%), and Proteobacteria (0.1%). Firmicutes and Bacteroidetes were present in all samples, while the other identified phyla were absent in at least one sample. Probiotic supplementation increased the relative abundance of Bacteroidetes and reduced the relative abundance of Actinobacteria compared to the control group (*p* < 0.05).

- Family Level—Sow Feces

At family level, thirty-one bacterial families were identified in total across all sow fecal samples. The top five families, based on relative abundance across all samples, accounted for 70.5% of the total abundance and included Ruminococcaceae (21.3%), Rikenellaceae (20.0%), Clostridiaceae (15.9%), Prevotellaceae (8.2%), and Muribaculaceae (5.1%). Ruminococcaceae, Rikenellaceae, Clostridiaceae, Prevotellaceae, Erysipelotrichaceae, Lachnospiraceae, and Oscillospiraceae were present in all samples, while the other identified families were absent in at least one sample. Probiotic supplementation increased the relative abundance of Rikenellaceae and reduced the relative abundance of Lactobacillaceae, Hungateiclostridiaceae, and Christensenellaceae compared to the control group (*p* < 0.05).

- Genus Level—Sow Feces

At genus level, seventy-two bacterial genera were identified in total across all sow fecal samples. The top five genera, based on relative abundance across all samples, accounted for 52.3% of the total abundance and included *Anaerocella* (16.1%), *Clostridium* (15.6%), *Sporobacter* (8.6%), *Prevotella* (7.2%), and *Duncaniella* (4.8%). *Anaerocella*, *Clostridium*, *Sporobacter*, *Oscillibacter*, and *Turicibacter* were present in all samples, while the other identified genera were absent in at least one sample. Probiotic supplementation increased the abundance of *Anaerocella* and *Sporobacter* and reduced the relative abundance of *Lactobacillus*, *Ruminococcus*, and *Christensenella* compared to the control group (*p* < 0.05).

#### 3.6.2. Offspring Microbial Analysis

Due to the limited creep intake, the effect of creep diet was not analyzed in the offspring microbial analysis. For the microbial analysis, sixteen samples (*n* = 8/maternal diet) of pigs in the same creep dietary group were utilized.

##### Absolute Quantification of *Escherichia coli* in Offspring Ileal Mucosa Associated Microbiota

The effect of maternal probiotic supplementation on the gene copy number of *Escherichia coli* in the offspring’s ileal mucosa-associated microbiota was analyzed via QPCR. Values are expressed as the logarithm (log) of the gene copy number.

There was no difference in the gene copy number of *Escherichia coli* in the ileal mucosa-associated microbiota of offspring from probiotic sows compared to offspring from control sows on a copy number per microliter of extracted DNA basis (3.4 vs. 4.3, SEM = 0.41) (*p* = 0.1365) or on a copy number per nanogram of DNA basis (0.5 vs. 1.42, SEM = 0.41) (*p* = 0.1271). Extracted DNA concentration was similar between offspring from probiotic sows and offspring from control sows (2.92 vs. 2.89, SEM = 0.03) (*p* = 0.3992).

##### 16S rRNA Microbial Analysis of Offspring Colonic Digesta

- Bacterial Richness and Diversity—Offspring Colonic Digesta

There was no difference in the beta diversity of the colonic digesta in offspring from control sows or offspring from probiotic sows based on a PERMANOVA analysis of Bray–Curtis dissimilarity (*p* < 0.05). The effects of maternal probiotic supplementation on the measures of alpha diversity in the piglet colonic digesta at weaning are presented in Table 12, respectively. Maternal probiotic supplementation had no effect on the Observed, Chao1, Shannon, Simpson, InvSimpson, or Fisher index measures of alpha diversity in the piglets’ colonic digesta at weaning (*p* > 0.05).

- Differential Microbial Abundance Analysis—Offspring Colonic Digesta

All data on differential bacterial abundances at phylum, family, and genus level in the offspring colonic digesta are presented in Table 13.

- Phylum Level—Offspring Colonic Digesta

At phylum level, seven bacterial phyla were identified in total across all piglet colonic digesta samples: Firmicutes (71.5%), Bacteroidetes (17.4%), Protobacteria (4.5%), Actinobacteria (4.0%), Tenericutes (0.7%), Fusobacteria (0.5%), and Synergistetes (0.2%). Only Firmicutes were present in all samples, while the other identified phyla were absent in at least one sample. Maternal probiotic supplementation increased the relative abundance of Firmicutes and reduced the relative abundance of Bacteroidetes and Proteobacteria in the offsprings colonic digesta at weaning compared to the control group (*p* < 0.05).

- Family Level—Offspring Colonic Digesta

At family level, thirty-one bacterial families were identified in total across all piglet colonic digesta samples. The top five families, based on relative abundance across all samples, accounted for 61.6% of the total abundance and included Lachnospiraceae (16.3%), Ruminococcaceae (16.0%), Prevotellaceae (11.7%), Lactobacillaceae (10.2%) and Eubacteriaceae (7.4%). Lachnospiraceae, Eubacteriaceae, and Acidaminococcaceae were present in all samples, while the other identified families were absent in at least one sample. Maternal probiotic supplementation increased the relative abundance of Lachnospiraceae, Hungateiclostridiaceae, and Coriobacteriaceae and reduced the relative abundance of Enterobacteriaceae, Oscillospiraceae, and Muribaculaceae in the offsprings colonic digesta at weaning compared to the control group (*p* < 0.05).

- Genus Level—Offspring Colonic Digesta

At genus level, sixty-three bacterial genera were identified in total across all piglet colonic digesta samples. The top five genera, based on relative abundance across all samples, accounted for 36.5% of the total abundance and included *Lactobacillus* (10.3%), *Prevotella* (10.2%), *Eubacterium* (7.9%), *Dorea* (4.3%), and *Oscillibacter* (3.8%). *Eubacterium* and *Phascolarctobacterium* were present in all samples, while the other identified genera were absent in at least one sample. Maternal probiotic supplementation increased the abundance of *Dorea, Anaerobacterium*, and *Sporobacter* and reduced the relative abundance of *Ruminococcus*, *Prevotellamassilia*, and *Faecalibacterium* in the offsprings colonic digesta at weaning compared to the control group (*p* < 0.05).

## 4. Discussion

In the present study, it was hypothesized that modulation of the sow’s microbiota would alter the microbial exposure of the offspring in early life, thereby influencing aspects of intestinal health pre-weaning. Additionally, it was hypothesized that increasing Trp in the piglets creep feed could positively modulate the microbiota composition and, via the action of both host and microbiota derived Trp metabolites, enhance intestinal health parameters. Indeed, maternal probiotic supplementation promoted distinct microbial communities in the sow feces at day 114 of gestation, increasing the relative abundance of *Anaerocella* and *Sporobacter* while decreasing *Lactobacillus*, *Ruminococcus*, and *Christensenella*. In the offspring colonic digesta, maternal probiotic supplementation led to an increase in the phylum Firmicutes, with notable increases in the genera *Dorea*, *Sporobacter*, and *Anaerobacterium* while reducing the potentially harmful phylum Proteobacteria, specifically the family Enterobacteriaceae. Furthermore, in the offspring, maternal probiotic supplementation enhanced duodenal morphology, modulated the gene expression in the ileum, reduced BCFA in the cecal digesta, and increased the total VFA and acetate concentration in the colonic digesta at weaning. However, there were no corresponding effects on offspring growth performance. The effect of the level of Trp in the piglet creep feed and the potential maternal × creep interaction may have been confounded by extremely limited creep intakes (~0.86 kg/litter), which potentially underpinned the minimal effects on performance or intestinal health parameters.

Given the significant contribution of the sow in the establishment of her offspring’s microbiota [15,16,17,18,19], maternal probiotic supplementation is considered a promising strategy to enhance the sow’s microbiota composition and consequently that of her offspring [22,23,24]. In the current study, supplementation with a probiotic blend of *Bacillus subtilis* and *Bacillus amyloliquefaciens* modulated the fecal microbial composition of sows on day 114 of gestation, creating distinct microbial communities in the probiotic sows compared to the control sows, as indicated by the significant Bray–Curtis dissimilarity. Probiotic supplementation increased the abundance of the phylum Bacteroidetes, within which the relative abundance of the family Rikenellaceae increased, and this change was further reflected at the genus level with an increase in *Anaerocella*. In ex situ studies, such as those conducted in anaerobic digesters and metrogenic reactors, *Anaerocella* are dominant producers of short-chain fatty acids (SCFA) [62,63]. Their role in the GIT microbiota could potentially lead to increases in SCFA production in sows, which would be a beneficial source of energy and positively influence intestinal health and immune function [64,65,66,67]. The genera *Sporobacter*, of the phylum Firmicutes, was also increased in the feces of probiotic sows. *Sporobacter* was positively correlated with apparent digestibility of neutral detergent fiber in finisher pigs though not in sows [68]. The role of *Sporobacter* in the GIT microbiota is not well documented in the literature, but it belongs to the family Ruminococcaceae, which is generally regarded as beneficial due to their role in polysaccharide fermentation and SCFA production [69,70]. Although *Sporobacter* increased in probiotic sows, there was a corresponding decrease in the relative abundance of *Ruminococcus* within the Ruminococcaceae family. Additionally, the relative abundance of the family Lactobacillaceae and the genus *Lactobacillus* decreased in probiotic sows. *Lactobacillus*, a beneficial SCFA-producing genus, is commonly included in probiotic formulations for maternal diets, where it has been associated with positive outcomes [71,72,73]. The reduction in *Lactobacillus* might be perceived as a negative effect; however, the impact of microbiota composition on health and performance is more likely driven by the collective interactions or community effect within the microbiota ecosystem rather than by a single genus.

Enteric colibacillosis is a prevalent condition in nursing and weaned pigs, resulting from the colonization of the small intestine by enterotoxigenic *Escherichia coli* (ETEC) strains. The ileal mucosa is recognized as an area where the relative abundance of *Escherichia coli* can be particularly high [74]. In the analysis of the copy number of *Escherichia coli* in the ileal mucosa, counts were similar across maternal groups. However, in the colonic digesta, there was a reduction in the phylum Proteobacteria and family Enterobacteriaceae, while there was a tendency for a reduction in the genus *Escherichia* in offspring from probiotic sows. Furthermore, in offspring from probiotic sows, there was an increase in the phylum Firmicutes, while within this phylum, there were increases in the families Lachnospiraceae and Hungateiclostridiaceae and in the genera *Dorea*, *Anaerobacterium*, and *Sporobacter* in the colonic digesta. In a study by Zhu et al. [75], *Dorea* was negatively correlated to fecal score and is therefore suggested to play a beneficial role in intestinal homeostasis. There was also a decrease in the phylum Bacteroidetes and, within this phylum, a decrease in the family Muribaculaceae and, at genus level, a reduction in *Prevotellamassilia*.

There were similarities in the differential abundance analysis between probiotic sows and their piglets compared to their respective controls, with an increase in Sporobacter and a reduction in Ruminococcus in probiotic groups. There were also some contrasting effects, with the family Hungateiclostridiaceae increased in probiotic sows but reduced in their piglets compared to the control. Unlike in the sow feces, overall, there were not distinct microbial communities in offspring from probiotic sows compared to offspring from control sows, as indicated by the absence of significance in the Bray–Curtis dissimilarity. Furthermore, the alpha diversity indices were similar in offspring from probiotic sows and offspring from control sows.

Short-chain fatty acid production is correlated to the properties of the dietary fiber [76,77] and the composition of the microbiota [78]. SCFA can be utilized as a substrate for epithelial cells, promote barrier function, maintain immune homeostasis, and positively modulate the microbiota [67,79,80,81]. Interestingly, offspring from probiotic sows exhibited increased total VFA and acetate concentrations in the colonic digesta compared to controls, suggesting enhanced microbial fermentation and the production of metabolites that could promote intestinal health. The increase in acetate concentrations in the colonic digesta may be linked to the increase in the genus *Dorea* in the offspring from probiotic sows, as *Dorea* sp. was positively correlated with acetate concentrations in broiler chickens [82]. Additionally, an in vitro fermentation analysis by Zhu La et al. [82] confirmed that *Dorea* sp. produced SCFAs, primarily consisting of acetate, and expressed genes key to the acetate synthesis pathway.

The morphology of the small intestine is often used as a key indicator of intestinal health. Increased VH suggests greater digestive and absorptive capacity [83,84], while increased CD can be an indication of increased epithelial turnover and villus renewal, potentially in response to villus damage and atrophy. VH/CD ratio serves as a useful measure of the balance between these two parameters. On commercial farms, abrupt weaning is frequently associated with undesirable changes in intestinal morphology, such as villus atrophy and crypt hyperplasia, leading to reductions in the VH/CD ratio and negative impacts on digestion and absorption [85,86]. Therefore, additives that promote positive intestinal morphology development could help mitigate the adverse effects of weaning stress on intestinal health. In the current study, maternal probiotic supplementation increased VH and VH/CD ratio and reduced CD in the duodenum, suggesting that maternal probiotic supplementation enhanced offspring digestive and absorptive capacity. Despite the significant improvement in duodenal morphology with maternal probiotic supplementation, there were minimal effects on the expression of immune, barrier defense, or on nutrient transporter genes in the duodenum. However, maternal probiotic supplementation did increase the expression of the *SOD2*, an enzyme that plays a key role in the defense against reactive oxygen species [87,88].

In the ileum of the offspring, *TLR2*, which is typically stimulated by microbial lipoproteins, was downregulated in offspring from probiotic sows compared to control sows. Barrier-related genes *CLDN3* and *MUC1* and antimicrobial peptide *DEFB1* were also downregulated. This could be interpreted as a negative effect, given the crucial role claudins play in barrier integrity [89], defecins play in host defense [90], and mucins play in the mucus barrier [91]. The expression of the antioxidant enzyme *SOD2*, which was upregulated in the duodenum of offspring from probiotic sows, was downregulated in the ileum of offspring from probiotic sows compared to control sows. Furthermore, the expression of *HTR4*, a serotonin receptor, was also downregulated in the ileum of pigs from probiotic sows compared to control sows. These genes are interconnected with immune response within the intestine [92,93,94,95], and it is possible that the changes in gene expression represent a form of adaptive response following immune stimulation in offspring from control sows.

In terms of performance parameters, maternal probiotic supplementation had no significant effects, with only a tendency for reduced feed intake in probiotic sows. However, the probiotic sows did have numerical increases in several reproductive and production traits: born alive, number of pigs weaned per litter, weaning weight, and litter weaning weight compared to control sows. In recent studies, supplementation with this specific probiotic blend in maternal sow diets improved birth weights [28] and promoted offspring weight gain and weaning weights, both when supplemented solely to the sow [29] and in combination with direct piglet supplementation [28,30]. Interestingly, the increase in number of pigs weaned per litter and total litter weaning weight with probiotic supplementation was greater in the current study than in the studies by Konieczka et al. [30] and Mazur-Kuśnirek et al. [28]. These studies included a considerably greater number of litters than the current study, so it is possible that with a larger sample size, the observed numerical differences in the current study might have reached statistical significance.

Given the low creep intakes achieved in this study, it is not surprising that the level of Trp in the creep feed had minimal effect on duodenum morphology or duodenal and ileal gene expression. The total pre-weaning creep intake was ~860 g/litter or ~72 g/pig. While pre-weaning creep intake can vary, this is considered very low. For context, Konieczka et al. [30] achieved [36,37] a total intake of 420 g/pig with similar litter size and weaning age as the current study, while Arnaud et al. [96] achieved 565 g/pig, although with a slightly larger litter size and an increase in weaning age by 1 day. The post-weaning period is a critical time of GIT vulnerability in pigs [97]. Increased post-weaning Trp supplementation can limit intestinal dysfunction in pigs challenged with lipopolysaccharide at 35 days post weaning [36,37] and with diquat at 7 days post weaning [38]. The immediate post-weaning period is a turbulent time for pigs due to increased immune challenges. Increasing pre-weaning Trp supplementation may serve as a proactive measure to prepare the pig, potentially limiting negative immune responses and intestinal dysfunction during this critical period, highlighting it as an area for future research, perhaps with an alternative delivery approach.

## 5. Conclusions

The effect of Trp levels in the offspring’s creep feed and the potential maternal × creep interaction were confounded by very low creep feed intakes, resulting in minimal impact on performance or intestinal health parameters. Maternal probiotic supplementation led to distinct microbial communities in the sow feces at day 114 of gestation, increasing the relative abundance of *Anaerocella* and *Sporobacter* while decreasing *Lactobacillus*, *Ruminococcus*, and *Christensenella*. In the offspring colonic digesta, maternal probiotic supplementation led to an increase in the phylum Firmicutes, with notable increases in the genera *Dorea*, *Sporobacter*, and *Anaerobacterium* while reducing the potentially harmful phylum Proteobacteria, specifically the family Enterobacteriaceae, with a tendency for a reduction in the genus *Escherichia*. Furthermore, in the offspring, maternal probiotic supplementation enhanced duodenal morphology, modulated the gene expression in the ileum, reduced BCFA in the cecal digesta, and increased the total VFA and acetate concentration in the colonic digesta at weaning. In conclusion, maternal supplementation with *Bacillus subtilis* and *Bacillus amyloliquefaciens* had moderate beneficial effects on the intestinal health of the offspring at weaning, and whether these translate to improved post-weaning performance requires further investigation.

## Figures and Tables

**Table 1:** Ingredient composition of gestation, lactation, and creep diets.

Ingredients (g/kg)	Gestation Sow Diet ^a^	Lactation Sow Diet ^a^	Creep		
			0.22% Trp ^b^	0.27% Trp ^b^	0.33% Trp ^b^
Wheat	-	380	472	472	472
Barley	750	250	100	100	100
Maize	-	-	120	120	120
Soyabean meal	90	170	-	-	-
Soya bean 50	-	-	90	90	90
Full-fat soya	-	80	90	90	90
Soycomil	-	-	30	30	30
Whey protein	-	-	40	40	40
Soya oil	12	25	30	30	30
Soya hulls	120	10	-	-	-
Beet pulp	4	10	-	-	-
Pollard	-	40	-	-	-
Vitamins and mineral premix ^c^	1.5	1.5	3	3	3
Salt	4	5	2	2	2
Monocalcium phosphate	6	8	4.2	4.2	4.2
Limestone	9	12	4.5	4.5	4.5
Lysine-HCL 78.8%	2.2	4	5.8	5.8	5.8
Methionine	0.6	1.3	2.5	2.5	2.5
Threonine	0.7	2.5	2.8	2.8	2.8
Tryptophan	0	0.7	0.2	0.7	1.2

^a^ Dietary groups: (1) basal diet; (2) basal diet supplemented with 400 g probiotic per ton of feed. ^b^ Calculated from the tabulated nutritional composition. ^c^ Vitamin and mineral premix (per kg diet): sow diets: 70 mg of Fe as FeSO_4_; 60 mg of Mn as MnO; 80 mg of Zn as ZnO; 15 mg of Cu as CuSO_4_; 0.6 mg of I as calcium iodate on a calcium sulphate/calcium carbonate carrier; 0.2 mg Se as sodium selenite; 3.4 mg of vitamin A as retinyl acetate; 25 mg of vitamin D3 as cholecalciferol; 100 mg of vitamin E as DL-α-tocopheryl acetate; 2 mg of vitamin K as phytylmenaquinone, 2 mg of vitamin B1 as thiamine; 5 mg of vitamin B2 as riboflavin; 3 mg of vitamin B6 as pyridoxine; 0.015 mg of vitamin B12 as cyanocobalamin; 12 mg of nicotinic acid; 10 mg of pantothenic acid; 500 mg of choline chloride; 0.02 mg of biotin; 5 mg of folic acid. Creep diets: 250 mg choline chloride; 140 mg Fe; 112.5 mg Zn; 47 mg Mn; 25 mg Cu; 0.6 mg I; 0.3 mg S; 12 mg nicotinic acid; 10 mg pantothenic acid; 67 mg tocopherol; 4 mg menaquinone; 2 mg riboflavin; 2 mg thiamine; 1.8 mg retinol; 0.025 mg cholecalciferol; 0.015 mg pyridoxine; 0.01 mg cyanocobalamin.

**Table 2:** Analysis of chemical composition of diets (g/kg unless otherwise stated).

Ingredients (g/kg)	Gestation Sow Diet ^a^	Lactation Sow Diet ^a^	Creep		
			0.22% Trp ^b^	0.27% Trp ^b^	0.33% Trp ^b^
Dry matter	870	870	900	880	900
Crude protein (N × 6.25)	141.5	170.3	180.5	177.0	182.5
Gross energy (MJ/kg)	15.91	15.95	16.56	16.84	16.82
Ash	50.5	52.6	50	60	50
Neutral detergent fiber	240.0	135.0	145.1	135.2	141.2
Crude oil	26.6	51.0	38.2	46.0	42.4
Arginine	9.3	11.0	10.7	11.4	10.6
Histidine	3.5	4.1	4.3	4.3	4.2
Isoleucine	5.1	7.2	7.4	7.8	7.5
Leucine	11.3	12.6	13.1	13.5	14.4
Lysine	7.5	11.3	13.9	14.0	14.1
Methionine	2.5	2.5	4.8	4.5	4.5
Phenylalanine	6.1	8.0	8.2	8.0	8.3
Threonine	5.7	6.9	8.9	8.5	9.1
Tryptophan	1.8	2.2	2.4	2.8	3.2
Valine	6.6	7.9	9.7	8.2	9.3

^a^ Dietary groups: (1) basal diet; (2) basal diet supplemented with 400 g probiotic per ton of feed. ^b^ Calculated from the tabulated nutritional composition [46].

**Table 3:** Oligonucleotide sequences of forward and reverse primers used for the quantification of *Escherichia coli* gene copy number.

Target Bacteria	Forward and Reverse Primers (5′-3′)	Amplicon Size (bp)
*Escherichia coli*	F: CATGCCGCGTGTATGAAGAA R: CGGGTAACGTCAATGAGCAAA	112

**Table 4:** Panel of porcine oligonucleotide primers used for QPCR.

Target Gene	Gene Name	Accession No.	Forward Primer (5′–3′) Reverse Primer (5′–3′)	Amplicon Length (bp)
Immune response				
*AHR*	Aryl Hydrocarbon Receptor	NM_001303026.1	F: GCAGCGCCAACATCACCT R: GGGATTGGCTTGACAGTTTTC	70
*DEFB1*	Beta Defensin 1	NM_214442.2	F: CCTGCCCGCTCTTCAACA R: GTCAGCGGATGCAGCACTT	69
*DEFB3*	Beta Defensin 3	XM_021074698.1	F: GCACGCCTTCCTATCCAGTCT R: GGCAAAGAGAAGGTAGTGGATCCT	72
*IL6*	Interleukin 6	NM_214399.1	F: GACAAAGCCACCACCCCTAA R: CTCGTTCTGTGACTGCAGCTTATC	69
*CXCL8*	C-X-C Motif Chemokine Ligand 8	NM_213867.1	F: TGCACTTACTCTTGCCAGAACTG R: CAAACTGGCTGTTGCCTTCTT	82
*IL10*	Interleukin 10	NM_214041.1	F: GCCTTCGGCCCAGTGAA R: AGAGACCCGGTCAGCAACAA	71
*IL22*	Interleukin 22	XM_021091968.1	F: GATGAGAGAGCGCTGCTACCTGG R: GAAGGACGCCACCTCCTGCATGT	112
*TLR2*	Toll Like Receptor 2	NM_213761.1	F: CATCTTCGTGCTTTCCGAGAAC R: AAAGAGACGGAAGTGGGAGAAGT	79
*TLR4*	Toll Like Receptor 4	NM_001293317.1	F: TGCATGGAGCTGAATTTCTACAA R: GATAAATCCAGCACCTGCAGTTC	140
*TNF*	Tumor Necrosis Factor	NM_214022.1	F: TGGCCCCTTGAGCATCA R: CGGGCTTATCTGAGGTTTGAG	68
Intestinal barrier				
*CLDN3*	Claudin 3	NM_001160075.1	F: GAGGGCCTGTGGATGAACTG R: GAGTCGTACACTTTGCACTGCAT	65
*TJP1*	Tight Junction Protein 1	XM_021098827.1	F: TGAGAGCCAACCATGTCTTGAA R: CTCAGACCCGGCTCTCTGTCT	76
Mucin				
*MUC1*	Mucin 1	XM_001926883.1	F: ACACCCATGGGCGCTATGT R: GCCTGCAGAAACCTGCTCAT	68
*MUC2*	Mucin 2	AK231524	F: CAACGGCCTCTCCTTCTCTGT R: GCCACACTGGCCCTTTGT	70
Nutrient transporters				
*Fatty acid transporters*				
*FABP2*	Fatty Acid-Binding Protein 2	NM_001031780.1	F: CAGCCTCGCAGACGGAACTGAA R: GTGTTCTGGGCTGTGCTCCAAGA	102
*Monosaccharide transporters*				
*SLC2A1 (GLUT1)*	Solute Carrier Family 2 Member 1	XM_021098317.1	F: TGCTCATCAACCGCAATGA R: GTTCCGCGCAGCTTCTTC	70
*SLC2A2 (GLUT2)*	Solute Carrier Family 2 Member 2	NM_001097417.1	F: CCAGGCCCCATCCCCTGGTT R: GCGGGTCCAGTTGCTGAATGC	96
*Peptide and amino acid transporters*				
*SLC15A1 (PEPT1)*	Solute Carrier Family 15 Member 1	NM_214347.1	F: GGATAGCCTGTACCCCAAGCT R: CATCCTCCACGTGCTTCTTGA	73
*SLC7A5*	Solute Carrier Family 7 Member 5	XR_002344446.1	F: CGGTCCTTTGCCAGAAGCT R: CCTTGGCTCCTGCTGCTTAT	63
*SLC7A6*	Solute Carrier Family 7 Member 6	XM_021094151.1	F: AGCGCGACAGAGCATCCT R: ACGTGTCTGTTTTGGCCAATT	66
*SLC7A7*	Solute Carrier Family 7 Member 7	NM_001110421.1	F: TGATTCATGTTGAGCGGTTCA R: ACAAGTAGATCAGCGCCATGAG	72
Oxidative status				
*SOD2*	Superoxide Dismutase 2	NM_214127.2	F: GCTTGTTCTAACCAGGATCCC R: TAATACGCATGCTCCCACAC	83
*NOX1*	NAPDH Oxidase 1	XM_003484140.3	F: AGCCATGCTGAGATCCCAAT R: TGCTTTATGGCAGGCTTTCA	68
Serotonin receptor				
*HTR4*	5-Hydroxytryptamine Receptor 4	NM_001001267.1	F: TGAGCGCTACCGAAGACCTT R: TTGACGGTTGTGGTTGAACAG	63
Reference Genes				
*ACTB*	Beta Actin	XM_001927228.1	F: GGACATCGGATACCCAAGGA R: AAGTTGGAAGGCCGGTTAATTT	71
*HMBS*	Hydroxymethylbilane Synthase	NM_001097412.1	F: CTGAACAAAGGTGCCAAGAACA R: GCCCCGCAGACCAGTTAGT	74
*H375A*	H3.3 histone A (H3-3A)	NM_213930.1	F: CATGGCTCGTACAAAGCAGA R: ACCAGGCCTGTAACGATGAG	136
*YWHAZ*	Tyrosine 3-Monooxygenase/Tryptophan 5-Monooxygenase Activation Protein Zeta	NM_001315726.1	F: GGACATCGGATACCCAAGGA R: AAGTTGGAAGGCCGGTTAATTT	71

**Table 5:** Effect of diet on sow and offspring performance pre-weaning (least square means with their standard errors).

Maternal Diet	Control			Probiotic			SEM	*p*-Value		
Creep Diet	0.22% Trp	0.27% Trp	0.33% Trp	0.22% Trp	0.27% Trp	0.33% Trp		M	C	M × C
Litter size (n)										
Born alive	13.88	14.13	14.50	15.63	14.38	15.25	1.00	0.269	0.805	0.749
Stillborn	1.63	1.38	0.63	0.63	0.63	1.88	0.49	0.678	0.878	0.052
Post-cross fostering	14.00	14.00	14.00	14.00	14.00	14.00	0.00	1.000	1.000	1.000
D7	13.38	13.25	13.13	13.38	13.13	13.25	0.35	0.607	0.980	0.159
D21	13.00	12.50	12.38	12.20	12.88	13.25	0.38	0.578	0.831	**0.049**
D26 (weaning)	13.00	12.00	12.00	12.00	12.75	13.25	0.48	0.399	0.873	0.058
Piglet mortality%	7.14	14.29	14.29	14.29	8.93	5.36	3.42	0.398	0.873	0.058
Piglet weight (kg)										
Birth	1.53	1.44	1.43	1.45	1.59	1.48	0.06	0.431	0.643	0.215
D7	2.70	2.58	2.35	2.50	2.57	2.80	0.16	0.607	0.980	0.159
D21	6.10	6.17	5.63	5.90	6.69	6.07	0.31	0.343	0.196	0.477
D26 (weaning)	7.37	7.48	6.93	7.58	7.41	7.16	0.38	0.710	0.486	0.931
Weaned litter weight (kg)	95.30	86.90	82.96	91.64	91.63	94.75	4.67	0.301	0.581	0.279
Piglet daily gain (kg)										
D0–7	0.17	0.16	0.13	0.14	0.14	0.19	0.02	0.929	0.917	0.075
D7–21	0.24	0.26	0.24	0.25	0.28	0.23	0.02	0.578	0.273	0.884
D21–26	0.25	0.26	0.26	0.23	0.28	0.22	0.01	0.349	0.191	0.173
D0–26	0.22	0.23	0.21	0.24	0.24	0.22	0.02	0.527	0.423	0.982
Total creep intake per litter (kg)	1.02	0.62	0.58	0.93	1.17	0.84	0.24	0.227	0.536	0.422
Sow backfat loss (mm)	−7.06	−6.00	−5.13	−6.94	−3.06	−3.94	1.20	0.156	0.071	0.503
Sow lactation feed intake (kg/day)	8.44	8.26	8.22	7.66	8.18	7.82	0.29	0.078	0.761	0.483
Gestation length (days)	117.38	117.38	118.50	117.13	117.25	117.00	0.54	0.164	0.605	0.380
Lactation length (days)	26.38	26.25	25.63	25.63	25.13	26.88	0.62	0.685	0.668	0.136
Weaning to service interval (days)	4.81	4.52	4.92	4.50	5.00	4.58	0.22	0.812	0.846	0.119

C, creep; D, day; M, maternal; Trp, tryptophan.

**Table 6:** Effect of diet on the duodenal morphology (least square means with their standard errors).

	Maternal		SEM	Creep			SEM	*p*-Value *	
	Control	Probiotic		0.22% Trp	0.27% Trp	0.33% Trp		M	C
VH (μm)	389.63	450.04	20.81	419.68	443.27	396.56	25.78	**0.047**	0.435
CD (μm)	138.80	110.62	5.45	126.62	125.28	122.23	6.75	**0.001**	0.891
VH/CD	2.89	4.17	0.22	3.50	3.72	3.36	0.28	**<0.001**	0.624

CD, crypt depth; Trp, tryptophan; VH, villus height; VH/CD, villus height to crypt depth ratio. * There was no maternal × creep interaction.

**Table 7:** Effect of diet on gene expression in the duodenum (least square means with their standard errors).

Maternal Diet		Control			Probiotic			SEM	*p*-value		
Creep Diet		0.22% Trp	0.27% Trp	0.33% Trp	0.22% Trp	0.27% Trp	0.33% Trp		M	C	M × C
Role	Gene										
Immune response	*AHR*	1.10	1.49	1.32	1.36	1.11	1.27	0.15	0.641	0.869	0.127
	*DEFB1*	0.74	0.90	0.63	0.80	1.18	0.84	0.19	0.247	0.229	0.841
	*DEFB3*	1.27	1.62	1.34	1.25	1.59	1.63	0.23	0.677	0.336	0.748
	*IL6*	1.11	1.08	1.08	1.10	1.04	1.84	0.21	0.168	0.125	0.103
	*CXCL8*	1.02	0.76	1.10	0.76	0.82	1.11	0.18	0.669	0.239	0.645
	*IL10*	1.37	1.09	1.07	1.01	0.88	1.76	0.18	0.788	0.078	**0.012**
	*IL22*	1.06	0.58	0.72	0.98	1.35	1.80	0.43	0.112	0.783	0.405
	*TLR2*	1.32	0.99	1.23	0.94	1.08	1.47	0.18	0.912	0.205	0.200
	*TLR4*	2.06	1.90	2.18	2.04	1.98	2.21	0.24	0.883	0.594	0.977
	*TNF*	1.15	1.27	1.20	1.24	1.31	1.46	0.19	0.405	0.783	0.835
Intestinal barrier	*CLDN3*	1.10	1.23	1.11	1.09	1.12	1.09	0.10	0.582	0.662	0.845
	*TJP1*	1.02	1.24	1.14	1.14	0.96	1.05	0.11	0.367	0.978	0.183
Mucin	*MUC1*	0.71	1.21	1.04	0.76	0.70	0.92	0.13	0.073	0.117	0.146
	*MUC2*	1.15	1.12	1.06	0.88	1.05	1.10	0.21	0.561	0.926	0.753
Nutrient transporters	*FABP2*	1.62	1.80	1.31	1.47	2.17	1.33	0.49	0.846	0.407	0.870
	*SLC2A1*	1.06	1.44	0.93	0.87	1.04	1.00	0.17	0.222	0.202	0.400
	*SLC2A2*	1.37	1.81	1.50	1.57	1.39	1.41	0.17	0.484	0.669	0.209
	*SLC15A1*	1.10	1.22	1.07	1.27	1.02	1.17	0.17	0.866	0.909	0.556
	*SLC7A5*	0.67	0.71	0.69	0.67	0.69	0.94	0.10	0.339	0.311	0.334
	*SLC7A6*	0.79	0.90	0.86	0.97	0.84	1.04	0.11	0.285	0.715	0.470
	*SLC7A7*	1.59	1.80	1.55	1.60	1.70	1.75	0.20	0.641	0.869	0.127
Oxidative status	*SOD2*	0.93	1.06	1.21	1.24	1.31	1.60	0.13	0.247	0.229	0.841
	*NOX1*	0.96	0.76	0.68	0.70	0.92	1.26	0.21	0.677	0.336	0.748
Serotonin receptor	*HTR4*	1.02	1.83	1.16	1.12	1.05	1.20	0.23	0.168	0.125	0.103

C, creep; M, maternal; Trp, tryptophan.

**Table 8:** Effect of diet on gene expression in the ileum (least square means with their standard errors).

Maternal Diet		Control			Probiotic			SEM	*p*-value		
Creep Diet		0.22% Trp	0.27% Trp	0.33% Trp	0.22% Trp	0.27% Trp	0.33% Trp		M	C	M x C
Role	Gene										
Immune response	*AHR*	1.14	1.23	1.03	0.93	0.99	1.13	0.08	0.054	0.586	0.054
	*DEFB1*	1.02	0.89	0.81	0.70	0.69	0.83	0.12	**0.045**	0.759	0.217
	*DEFB3*	0.93	1.16	1.00	0.94	1.08	0.94	0.09	0.578	0.127	0.899
	*IL6*	1.31	1.63	1.22	1.27	1.45	1.45	0.18	0.980	0.386	0.540
	*CXCL8*	0.79	0.74	0.88	0.63	0.53	0.70	0.16	0.149	0.617	0.984
	*IL10*	1.38	1.46	1.09	0.90	1.05	1.41	0.18	0.185	0.738	**0.044**
	*IL22*	1.03	0.89	0.79	0.90	0.68	1.19	0.20	0.904	0.554	0.288
	*TLR2*	0.98	0.92	1.03	0.70	0.69	0.70	0.11	**0.003**	0.850	0.887
	*TLR4*	0.90	1.13	1.12	1.01	0.92	1.11	0.16	0.760	0.662	0.622
	*TNF*	1.43	1.35	1.13	1.25	1.61	1.81	0.34	0.381	0.899	0.459
Intestinal barrier	*CLDN3*	0.95	0.88	1.15	0.61	0.46	0.67	0.17	**0.003**	0.343	0.920
	*TJP1*	0.95	0.98	1.01	0.91	1.00	0.99	0.05	0.774	0.266	0.803
Mucin	*MUC1*	0.97	0.84	0.78	0.61	0.56	0.61	0.10	**0.003**	0.598	0.685
	*MUC2*	0.84	0.85	0.63	0.45	0.61	0.96	0.17	0.490	0.692	0.100
Nutrient transporters	*SLC7A5*	1.13	1.27	0.89	1.12	1.43	1.56	0.14	**0.025**	0.321	0.055
	*SLC7A6*	1.06	1.25	0.97	0.98	1.09	1.12	0.07	0.648	0.125	0.128
	*SLC7A7*	1.06	0.93	1.13	0.76	0.62	0.66	0.19	**0.031**	0.760	0.893
Oxidative status	*SOD2*	0.97	0.94	0.86	0.73	0.67	0.77	0.08	**0.007**	0.855	0.571
	*NOX1*	1.27	1.33	0.93	0.93	0.99	1.17	0.11	0.108	0.636	**0.016**
Serotonin receptor	*HTR4*	0.79	1.00	0.97	0.77	0.64	0.60	0.15	**0.050**	0.956	0.460

C, creep; M, maternal; Trp, tryptophan.

**Table 9:** Effect of diet on volatile fatty acid concentrations in the offsprings cecal and colonic digesta at weaning (least square means with their standard errors).

	Maternal Diet		SEM	Creep Diet			SEM	*p*-Value *	
	Control	Probiotic		0.22% Trp	0.27% Trp	0.33% Trp		Maternal	Creep
Cecum (mmol/g digesta)									
Total VFA	176.22	187.71	23.98	173.28	186.15	186.49	28.05	0.708	0.926
Acetate	93.28	122.50	16.18	104.34	105.55	112.46	18.92	0.152	0.938
Propionate	47.15	42.64	7.03	38.92	51.47	44.31	8.23	0.616	0.537
Butyrate	18.67	15.41	2.05	17.36	17.44	16.33	2.40	0.217	0.922
Iso-butyrate	5.71	1.77	0.76	3.69	3.51	4.02	0.89	**<0.001**	0.901
Iso-valerate	6.63	3.17	0.72	4.54	4.67	5.49	0.84	**0.001**	0.642
Valerate	5.67	2.22	0.54	4.44	3.52	3.88	0.59	**<0.001**	0.569
Branch-chain fatty acids ^a^	18.01	7.16	1.51	12.67	11.69	13.39	1.76	**<0.001**	0.756
Colon (mmol/g digesta)									
Total VFA	104.15	151.19	14.00	155.32	115.38	112.31	17.85	**0.018**	0.155
Acetate	55.37	89.60	8.63	88.95	47.71	60.79	10.79	**0.005**	0.151
Propionate	27.34	33.40	3.38	37.46	27.83	25.82	4.31	0.193	0.117
Butyrate	10.58	11.27	1.23	13.70	9.26	9.80	1.57	0.679	0.089
Iso-butyrate	2.87	3.30	0.31	3.59	2.78	2.88	0.40	0.309	0.271
Iso-valerate	3.53	4.32	0.40	4.54	3.45	3.79	0.51	0.138	0.265
Valerate	4.18	5.05	0.57	6.09 ^x^	3.79 ^y^	3.96 ^y^	0.71	0.260	**0.041**
Branch-chain fatty acids ^a^	10.61	12.38	1.23	13.32	10.55	10.63	1.60	0.283	0.344

Trp, tryptophan; VFA, volatile fatty acid. ^a^ Branch-chain fatty acids were calculated by the sum of iso-butyrate, iso-valerate, and valerate. ^x y^ mean values within a row with different superscript letters were significantly different. * There was no maternal × creep interaction.

**Table 10:** The effect of probiotic supplementation on measures of alpha diversity in the sow feces at D114 of gestation (least square means with their standard errors).

	Control	Probiotic	SEM	*p*-Value
Observed	70.09	71.83	6.22	0.842
Chao1	70.09	71.83	6.22	0.842
Shannon	3.71	3.67	0.08	0.730
Simpson	0.96	0.95	0.01	0.146
InvSimpson	27.76	23.44	2.28	0.187
Fisher	12.02	12.20	1.35	0.926

A total of 12 replicates were used per dietary group.

**Table 11:** The effect of probiotic supplementation on the % bacterial abundance at phylum, family, and genus level in the sow feces at D114 of gestation (least square means with their standard errors).

				Control	Probiotic	SEM	*p*-Value
**Phylum**							
Bacteroidetes *				27.11	33.34	1.67	**0.011**
Actinobacteria *				1.22	0.44	0.32	**0.050**
**Phylum**		**Family**					
Bacteroidetes *		Rikenellaceae		17.55	22.50	1.37	**0.013**
Firmicutes *		Lactobacillaceae		6.24	3.13	0.72	**0.002**
Firmicutes *		Oscillospiraceae		5.06	3.42	0.65	0.066
Firmicutes *		Christensenellaceae		3.04	1.63	0.50	**0.037**
Firmicutes *		Hungateiclostridiaceae		1.64	0.56	0.37	**0.025**
**Phylum**	**Family**		**Genus**				
Bacteroidetes *	Rikenellaceae		*Anaerocella*	14.14	17.98	1.22	**0.028**
Firmicutes *	Ruminococcaceae		*Sporobacter*	7.16	9.97	0.91	**0.029**
Firmicutes *	Lactobacillaceae		*Lactobacillus*	6.28	3.09	0.72	**0.002**
Firmicutes *	Oscillospiraceae		*Oscillibacter*	5.04	3.34	0.07	0.056
Firmicutes *	Ruminococcaceae		*Ruminococcus*	4.66	2.91	0.62	**0.039**
Firmicutes *	Christensenellaceae		*Christensenella*	2.94	1.60	0.50	**0.043**
Firmicutes *	Hungateiclostridiaceae		*Ruminiclostridium*	1.05	0.43	0.32	0.100
Firmicutes *	Lachnospiraceae		*Coprococcus*	0.08	0.61	0.23	0.073
Firmicutes *	Lachnospiraceae		*Eisenbergiella*	0.57	0.08	0.22	0.087

A total of 12 replicates were used per dietary group. * Also known as on NCBI Taxonomy Browser: Bacteroidetes (Bacteroidota); Actinobacteria (Actinobacterota); Firmicutes (Bacillota).

**Table 12:** The effect of maternal probiotic supplementation on measures of alpha diversity in piglet colonic digesta at weaning (least square means with their standard errors).

	Control	Probiotic	SEM	*p*-Value
Observed	80.00	73.25	7.54	0.537
Chao1	80.00	73.25	7.54	0.537
Shannon	3.89	3.81	0.10	0.558
Simpson	0.97	0.97	0.00	0.944
InvSimpson	34.95	32.08	3.77	0.598
Fisher	12.63	11.35	1.44	0.539

A total of eight replicates were used per dietary group.

**Table 13:** The effect of probiotic supplementation on the % bacterial abundance at phylum, family, and genus level in the offspring colonic digesta on the day of weaning (least square means with their standard errors).

				Control	Probiotic	SEM	*p*-Value
**Phylum**							
Firmicutes *				66.12	76.96	3.10	**0.023**
Bacteroidetes *				21.51	13.36	1.64	**0.002**
Proteobacteria				6.27	2.81	0.89	**0.007**
**Phylum**		**Family**					
Firmicutes *		Lachnospiraceae		13.99	18.57	1.53	**0.040**
Proteobacteria *		Enterobacteriaceae		6.21	2.94	0.88	**0.010**
Firmicutes *		Oscillospiraceae		4.82	2.82	0.78	0.062
Firmicutes *		Hungateiclostridiaceae		2.34	4.86	0.78	**0.021**
Actinobacteria *		Coriobacteriaceae		0.81	1.96	0.50	0.079
Bacteroidetes *		Muribaculaceae		2.20	0.41	0.52	**0.014**
**Phylum**	**Family**		**Genus**				
Firmicutes *	Lachnospiraceae		*Dorea*	2.95	5.60	0.84	**0.025**
Firmicutes *	Oscillospiraceae		*Oscillibacter*	4.80	2.73	0.77	0.054
Firmicutes *	Ruminococcaceae		*Sporobacter*	1.53	3.17	0.44	**0.050**
Firmicutes *	Hungateiclostridiaceae		*Anaerobacterium*	0.65	4.02	0.71	**0.002**
Firmicutes *	Ruminococcaceae		*Papillibacter*	1.28	2.70	0.58	0.070
Firmicutes *	Ruminococcaceae		*Ruminococcus*	2.65	0.61	0.58	**0.011**
Bacteroidetes *	Prevotellaceae		*Prevotellamassilia*	2.88	0.22	0.60	**0.005**
Actinobacteria *	Coriobacteriaceae		*Colllinsella*	0.91	1.99	0.50	0.100
Firmicutes *	Ruminococcaceae		*Faecalibacterium*	2.25	0.44	0.53	**0.014**
Proteobacteria *	Enterobacteriaceae		*Escherichia*	0.83	0.08	0.31	0.091

A total of eight replicates were used per dietary group. * Also known as on NCBI Taxonomy Browser: Bacteroidetes (Bacteroidota); Actinobacteria (Actinobacterota); Firmicutes (Bacillota); Proteobacteria (Pseudomonadota).

## Data Availability

All data presented and/or analyzed in this study are available on request from the corresponding author.

## References

[1] Déru V., Bouquet A., Zemb O., Blanchet B., De Almeida M.L., Cauquil L., Carillier-Jacquin C., Gilbert H. (2022). Genetic relationships between efficiency traits and gut microbiota traits in growing pigs being fed with a conventional or a high-fiber diet. J. Anim. Sci..

[2] Wang X., Tsai T., Deng F., Wei X., Chai J., Knapp J., Apple J., Maxwell C.V., Lee J.A., Li Y. (2019). Longitudinal investigation of the swine gut microbiome from birth to market reveals stage and growth performance associated bacteria. Microbiome.

[3] Argüello H., Estellé J., Leonard F.C., Crispie F., Cotter P.D., O’sullivan O., Lynch H., Walia K., Duffy G., Lawlor P.G. (2019). Influence of the Intestinal Microbiota on Colonization Resistance to *Salmonella* and the Shedding Pattern of Naturally Exposed Pigs. mSystems.

[4] Luppi A., Gibellini M., Gin T., Vangroenweghe F., Vandenbroucke V., Bauerfeind R., Bonilauri P., Labarque G., Hidalgo Á. (2016). Prevalence of virulence factors in enterotoxigenic *Escherichia coli* isolated from pigs with post-weaning diarrhoea in Europe. Porc. Health Manag..

[5] Van Breda L.K., Dhungyel O.P., Ginn A.N., Iredell J.R., Ward M.P. (2017). Pre- and post-weaning scours in southeastern Australia: A survey of 22 commercial pig herds and characterisation of *Escherichia coli* isolates. PLoS ONE.

[6] Jacobson M. (2022). On the Infectious Causes of Neonatal Piglet Diarrhoea—A Review. Vet. Sci..

[7] Thompson C.L., Wang B., Holmes A.J. (2008). The immediate environment during postnatal development has long-term impact on gut community structure in pigs. ISME J..

[8] Schmidt B., Mulder I.E., Musk C.C., Aminov R.I., Lewis M., Stokes C.R., Bailey M., Prosser J.I., Gill B.P., Pluske J.R. (2011). Establishment of normal gut microbiota is compromised under excessive hygiene conditions. PLoS ONE.

[9] Merrifield C.A., Lewis M.C., Berger B., Cloarec O., Heinzmann S.S., Charton F., Krause L., Levin N.S., Duncker S., Mercenier A. (2016). Neonatal environment exerts a sustained influence on the development of the intestinal microbiota and metabolic phenotype. ISME J..

[10] Sprockett D., Fukami T., Relman D.A. (2018). Role of priority effects in the early-life assembly of the gut microbiota. Nat. Rev. Gastroenterol. Hepatol..

[11] Schokker D., Zhang J., Vastenhouw S.A., Heilig H.G.H.J., Smidt H., Rebel J.M.J., Smits M.A. (2015). Long-lasting effects of early-life antibiotic treatment and routine animal handling on gut microbiota composition and immune system in pigs. PLoS ONE.

[12] Mulder I.E., Schmidt B., Lewis M., Delday M., Stokes C.R., Bailey M., Aminov R.I., Gill B.P., Pluske J.R., Mayer C.-D. (2011). Restricting Microbial Exposure in Early Life Negates the Immune Benefits Associated with Gut Colonization in Environments of High Microbial Diversity. PLoS ONE.

[13] Arnal M.-E., Zhang J., Messori S., Bosi P., Smidt H., Lallès J.-P. (2014). Early changes in microbial colonization selectively modulate intestinal enzymes, but not inducible heat shock proteins in young adult Swine. PLoS ONE.

[14] Xiang Q., Wu X., Pan Y., Wang L., Cui C., Guo Y., Zhu L., Peng J., Wei H. (2020). Early-Life Intervention Using Fecal Microbiota Combined with Probiotics Promotes Gut Microbiota Maturation, Regulates Immune System Development, and Alleviates Weaning Stress in Piglets. Int. J. Mol. Sci..

[15] Chen X., Xu J., Ren E., Su Y., Zhu W. (2018). Co-occurrence of early gut colonization in neonatal piglets with microbiota in the maternal and surrounding delivery environments. Anaerobe.

[16] Liu H., Zeng X., Zhang G., Hou C., Li N., Yu H., Shang L., Zhang X., Trevisi P., Yang F. (2019). Maternal Breast Milk and Fecal Microbes Guide the Spatiotemporal Development of Mucosa-Associated Microbiota and Barrier Function in the Neonatal Gut. BMC Biol..

[17] Lim J.-A., Cha J., Choi S., Kim J.-H., Kim D. (2023). Early Colonization of the Intestinal Microbiome of Neonatal Piglets Is Influenced by the Maternal Microbiome. Animals.

[18] Liu S., Zhang Z., Ma L. (2023). A Review Focusing on Microbial Vertical Transmission during Sow Pregnancy. Veter- Sci..

[19] Adams S., Knapp J.P., Neujahr A., Burkey T., Miller P.S., Fernando S.C. (2023). 276 Investigating the Colonization History of Early-Life Microbiome of Piglets. J. Anim. Sci..

[20] Quan J., Xu C., Ruan D., Ye Y., Qiu Y., Wu J., Zhou S., Luan M., Zhao X., Chen Y. (2023). Composition, function, and timing: Exploring the early-life gut microbiota in piglets for probiotic interventions. J. Anim. Sci. Biotechnol..

[21] Kiernan D.P., O’Doherty J.V., Sweeney T. (2023). The effect of maternal probiotic or synbiotic supplementation on sow and offspring microbiome, health, and performance. Animals.

[22] Kritas S.K., Marubashi T., Filioussis G., Petridou E., Christodoulopoulos G., Burriel A.R., Tzivara A., Theodoridis A., Pískoriková M. (2015). Reproductive performance of sows was improved by administration of a sporing bacillary probiotic (*Bacillus subtilis* C-3102). J. Anim. Sci..

[23] Han L., Azad A.K., Huang P., Wang W., Zhang W., Blachier F., Kong X. (2022). Maternal Supplementation with Different Probiotic Mixture from Late Pregnancy to Day 21 Postpartum: Consequences for Litter Size, Plasma and Colostrum Parameters, and Fecal Microbiota and Metabolites in Sows. Front. Vet. Sci..

[24] Liu H., Wang S., Zhang D., Wang J., Zhang W., Wang Y., Ji H. (2020). Effects of dietary supplementation with Pediococcus acidilactici ZPA017 on reproductive performance, fecal microbial flora and serum indices in sows during late gestation and lactation. Asian-Australas. J. Anim. Sci..

[25] Crespo-Piazuelo D., Gardiner G.E., Ranjitkar S., Bouwhuis M.A., Ham R., Phelan J.P., Marsh A., Lawlor P.G. (2022). Maternal supplementation with *Bacillus altitudinis* spores improves porcine offspring growth performance and carcass weight. Br. J. Nutr..

[26] Baker A.A., Davis E., Spencer J.D., Moser R., Rehberger T. (2013). The effect of a Bacillus-based direct-fed microbial supplemented to sows on the gastrointestinal microbiota of their neonatal piglets. J. Anim. Sci..

[27] Larsen N., Thorsen L., Kpikpi E.N., Stuer-Lauridsen B., Cantor M.D., Nielsen B., Brockmann E., Derkx P.M.F., Jespersen L. (2014). Characterization of Bacillus spp. strains for use as probiotic additives in pig feed. Appl. Microbiol. Biotechnol..

[28] Mazur-Kuśnirek M., Lipiński K., Jørgensen J.N., Hansen L.H.B., Antoszkiewicz Z., Zabielski R., Konieczka P. (2023). The Effect of a *Bacillus*-Based Probiotic on Sow and Piglet Performance in Two Production Cycles. Animals.

[29] Barbosa A.M.S., Carvalho M.P.S., Naves L.d.P., da Motta S.A.B., Chaves R.F., Resende M., de Lima D., Hansen L.H.B., Cantarelli V.d.S. (2024). Performance and Health Parameters of Sows and Their Litters Using a Probiotic Supplement Composed of *Bacillus subtilis* 541 and *Bacillus amyloliquefaciens* 516. Animals.

[30] Konieczka P., Ferenc K., Jørgensen J.N., Hansen L.H., Zabielski R., Olszewski J., Gajewski Z., Mazur-Kuśnirek M., Szkopek D., Szyryńska N. (2023). Feeding Bacillus-based probiotics to gestating and lactating sows is an efficient method for improving immunity, gut functional status and biofilm formation by probiotic bacteria in piglets at weaning. Anim. Nutr..

[31] Saladrigas-García M., Solà-Oriol D., López-Vergé S., D’angelo M., Collado M.C., Nielsen B., Faldyna M., Pérez J.F., Martín-Orúe S.M. (2022). Potential effect of two *Bacillus* probiotic strains on performance and fecal microbiota of breeding sows and their piglets. J. Anim. Sci..

[32] Kiernan D.P., O’doherty J.V., Ryan M.T., Sweeney T. (2025). Effects of Maternal Probiotics and Piglet Dietary Tryptophan Level on Gastric Function Pre- and Post-Weaning. Agriculture.

[33] Li X., Zhang Z.-H., Zabed H.M., Yun J., Zhang G., Qi X. (2021). An Insight into the Roles of Dietary Tryptophan and Its Metabolites in Intestinal Inflammation and Inflammatory Bowel Disease. Mol. Nutr. Food Res..

[34] Bessede A., Gargaro M., Pallotta M.T., Matino D., Servillo G., Brunacci C., Bicciato S., Mazza E.M.C., Macchiarulo A., Vacca C. (2014). Aryl hydrocarbon receptor control of a disease tolerance defence pathway. Nature.

[35] Lamas B., Natividad J.M., Sokol H. (2018). Aryl hydrocarbon receptor and intestinal immunity. Mucosal Immunol..

[36] Liu G., Lu J., Sun W., Jia G., Zhao H., Chen X., Kim I.H., Zhang R., Wang J. (2022). Tryptophan supplementation enhances intestinal health by improving gut barrier function, alleviating inflammation, and modulating intestinal microbiome in lipopolysaccharide-challenged piglets. Front. Microbiol..

[37] Liu G., Tao J., Lu J., Jia G., Zhao H., Chen X., Tian G., Cai J., Zhang R., Wang J. (2022). Dietary Tryptophan Supplementation Improves Antioxidant Status and Alleviates Inflammation, Endoplasmic Reticulum Stress, Apoptosis, and Pyroptosis in the Intestine of Piglets after Lipopolysaccharide Challenge. Antioxidants.

[38] Liu J., Zhang Y., Li Y., Yan H., Zhang H. (2019). L-Tryptophan Enhances Intestinal Integrity in Diquat-Challenged Piglets Associated with Improvement of Redox Status and Mitochondrial Function. Animals.

[39] Zhang L., Nichols R.G., Correll J., Murray I.A., Tanaka N., Smith P.B., Hubbard T.D., Sebastian A., Albert I., Hatzakis E. (2015). Persistent organic pollutants modify gut microbiota–host metabolic homeostasis in mice through aryl hydrocarbon receptor activation. Environ. Health Perspect..

[40] Tossou M.C.B., Liu H., Bai M., Chen S., Cai Y., Duraipandiyan V., Liu H., Adebowale T.O., Al-Dhabi N.A., Long L. (2016). Effect of High Dietary Tryptophan on Intestinal Morphology and Tight Junction Protein of Weaned Pig. BioMed Res. Int..

[41] Beaumont M., Roura E., Lambert W., Turni C., Michiels J., Chalvon-Demersay T. (2022). Selective nourishing of gut microbiota with amino acids: A novel prebiotic approach?. Front. Nutr..

[42] Kiernan D.P., O’doherty J.V., Sweeney T. (2023). The effect of prebiotic supplements on the gastrointestinal microbiota and associated health parameters in the pig. Animals.

[43] Liang H., Dai Z., Liu N., Ji Y., Chen J., Zhang Y., Yang Y., Li J., Wu Z., Wu G. (2018). Dietary L-Tryptophan Modulates the Structural and Functional Composition of the Intestinal Microbiome in Weaned Piglets. Front. Microbiol..

[44] Rao Z., Li J., Shi B., Zeng Y., Liu Y., Sun Z., Wu L., Sun W., Tang Z. (2021). Dietary Tryptophan Levels Impact Growth Performance and Intestinal Microbial Ecology in Weaned Piglets via Tryptophan Metabolites and Intestinal Antimicrobial Peptides. Animals.

[45] Chen C., Hu H., Li Z., Qi M., Qiu Y., Hu Z., Feng F., Tang W., Diao H., Sun W. (2024). Dietary tryptophan improves growth and intestinal health by promoting the secretion of intestinal β-defensins against enterotoxigenic *Escherichia coli* F4 in weaned piglets. J. Nutr. Biochem..

[46] Sauvant D., Perez J.-M., Tran G. (2023). Tables of Composition and Nutritional Value of Feed Materials: Pigs, Poultry, Cattle, Sheep, Goats, Rabbits, Horses and Fish.

[47] Caporaso J.G., Kuczynski J., Stombaugh J., Bittinger K., Bushman F.D., Costello E.K., Fierer N., Gonzalez Peña A., Goodrich J.K., Gordon J.I. (2010). QIIME allows analysis of high-throughput community sequencing data. Nat. Methods.

[48] Magoč T., Salzberg S.L. (2011). FLASH: Fast length adjustment of short reads to improve genome assemblies. Bioinformatics.

[49] Edgar R.C., Haas B.J., Clemente J.C., Quince C., Knight R. (2011). UCHIME improves sensitivity and speed of chimera detection. Bioinformatics.

[50] Rognes T., Flouri T., Nichols B., Quince C., Mahé F. (2016). VSEARCH: A versatile open source tool for metagenomics. PeerJ.

[51] Eren A.M., Maignien L., Sul W.J., Murphy L.G., Grim S.L., Morrison H.G., Sogin M.L. (2013). Oligotyping: Differentiating between closely related microbial taxa using 16S rRNA gene data. Methods Ecol. Evol..

[52] Eren A.M., Morrison H.G., Lescault P.J., Reveillaud J., Vineis J.H., Sogin M.L. (2015). Minimum entropy decomposition: Unsupervised oligotyping for sensitive partitioning of high-throughput marker gene sequences. ISME J..

[53] Angly F.E., Dennis P.G., Skarshewski A., Vanwonterghem I., Hugenholtz P., Tyson G.W. (2014). CopyRighter: A rapid tool for improving the accuracy of microbial community profiles through lineage-specific gene copy number correction. Microbiome.

[54] Dowley A., Sweeney T., Conway E., Vigors S., Yadav S., Wilson J., Gabrielli W., O’doherty J.V. (2021). Effects of Dietary Supplementation with Mushroom or Vitamin D_2_-Enriched Mushroom Powders on Gastrointestinal Health Parameters in the Weaned Pig. Animals.

[55] Clarke L.C., Sweeney T., Curley E., Gath V., Duffy S.K., Vigors S., Rajauria G., O’Doherty J.V. (2018). Effect of β-glucanase and β-xylanase enzyme supplemented barley diets on nutrient digestibility, growth performance and expression of intestinal nutrient transporter genes in finisher pigs. Anim. Feed. Sci. Technol..

[56] Dowley A., O’doherty J.V., Mukhopadhya A., Conway E., Vigors S., Maher S., Ryan M.T., Sweeney T. (2022). Maternal Supplementation With a Casein Hydrolysate and Yeast Beta-glucan From Late Gestation Through Lactation Improves Gastrointestinal Health of Piglets at Weaning. Sci. Rep..

[57] Hu Z., Feng L., Jiang Q., Wang W., Tan B., Tang X., Yin Y. (2023). Intestinal tryptophan metabolism in disease prevention and swine production. Anim. Nutr..

[58] Fabà L., de Groot N., Ramis G., Cabrera-Gómez C.G., Doelman J. (2022). Serotonin receptors and their association with the immune system in the gastrointestinal tract of weaning piglets. Porc. Health Manag..

[59] Ryan M.T., O’doherty J.V., Sweeney T. (2024). A Transcriptomic Evaluation of Neuroactive Receptors in the Colon of a Dextran Sodium Sulphate Pig Model of Colitis. Nutraceuticals.

[60] Harrell F.E., Harrell M.F.E. (2019). Package ‘hmisc’. CRAN2018.

[61] Barret Schloerke D.C., Joseph L., Francois B., Moritz M., Edwin T., Amos E., Ott T., Jason C. (2024). GGally: Extension to ‘ggplot2’. R Package Version 2.2.1. https://archive.softwareheritage.org/browse/directory/16636441710e633dfb96108e0b723390a0740073/?origin_url=https://hal.archives-ouvertes.fr/halshs-03354562&release=HEAD&snapshot=a670af2ebeeeae775b0c9573c9571ffaca8af228.

[62] Abe K., Ueki A., Ohtaki Y., Kaku N., Watanabe K., Ueki K. (2012). Anaerocella delicata gen. nov., sp. nov., a strictly anaerobic bacterium in the phylum Bacteroidetes isolated from a methanogenic reactor of cattle farms. J. Gen. Appl. Microbiol..

[63] Du X., Zhang Y., Ma Y.-W., Feng S.-X., Zhang Y.-X., Kou H.-J., Sun Y. (2023). The synergistic effect of chemical oxidation and microbial activity on improving volatile fatty acids (VFAs) production during the animal wastewater anaerobic digestion process treated with persulfate/biochar. Sci. Total Environ..

[64] Donohoe D.R., Garge N., Zhang X., Sun W., O’Connell T.M., Bunger M.K., Bultman S.J. (2011). The Microbiome and Butyrate Regulate Energy Metabolism and Autophagy in the Mammalian Colon. Cell Metab..

[65] Kien C.L., Blauwiekel R., Bunn J.Y., Jetton T.L., Frankel W.L., Holst J.J. (2007). Cecal infusion of butyrate increases intestinal cell proliferation in piglets. J. Nutr..

[66] Zeng X., Sunkara L.T., Jiang W., Bible M., Carter S., Ma X., Qiao S., Zhang G. (2013). Induction of Porcine Host Defense Peptide Gene Expression by Short-Chain Fatty Acids and Their Analogs. PLoS ONE.

[67] Singh N., Gurav A., Sivaprakasam S., Brady E., Padia R., Shi H., Thangaraju M., Prasad P.D., Manicassamy S., Munn D.H. (2014). Activation of Gpr109a, Receptor for Niacin and the Commensal Metabolite Butyrate, Suppresses Colonic Inflammation and Carcinogenesis. Immunity.

[68] Niu Q., Li P., Hao S., Kim S.W., Du T., Hua J., Huang R. (2019). Characteristics of Gut Microbiota in Sows and Their Relationship with Apparent Nutrient Digestibility. Int. J. Mol. Sci..

[69] Yang J., Martínez I., Walter J., Keshavarzian A., Rose D.J. (2013). In vitro characterization of the impact of selected dietary fibers on fecal microbiota composition and short chain fatty acid production. Anaerobe.

[70] Biddle A., Stewart L., Blanchard J., Leschine S. (2013). Untangling the Genetic Basis of Fibrolytic Specialization by Lachnospiraceae and Ruminococcaceae in Diverse Gut Communities. Diversity.

[71] Betancur C., Martínez Y., Tellez-Isaias G., Castillo R., Ding X. (2021). Effect of oral administration with Lactobacillus plantarum CAM6 strain on sows during gestation-lactation and the derived impact on their progeny performance. Mediat. Inflamm..

[72] Wang G., Wang X., Ma Y., Cai S., Yang L., Fan Y., Zeng X., Qiao S. (2022). Lactobacillus reuteri improves the development and maturation of fecal microbiota in piglets through mother-to-infant microbe and metabolite vertical transmission. Microbiome.

[73] Gao T., Li R., Hu L., Hu Q., Wen H., Zhou R., Yuan P., Zhang X., Huang L., Zhuo Y. (2024). Probiotic Lactobacillus rhamnosus GG improves insulin sensitivity and offspring survival via modulation of gut microbiota and serum metabolite in a sow model. J. Anim. Sci. Biotechnol..

[74] Kelly J., Daly K., Moran A.W., Ryan S., Bravo D., Shirazi-Beechey S.P. (2017). Composition and diversity of mucosa-associated microbiota along the entire length of the pig gastrointestinal tract; dietary influences. Environ. Microbiol..

[75] Zhu J., Sun Y., Ma L., Chen Q., Hu C., Yang H., Hong Q., Xiao Y. (2024). Comparative analysis of fecal microbiota between diarrhea and non-diarrhea piglets reveals biomarkers of gut microbiota associated with diarrhea. Anim. Nutr..

[76] Zhao J., Bai Y., Zhang G., Liu L., Lai C. (2020). Relationship between Dietary Fiber Fermentation and Volatile Fatty Acids’ Concentration in Growing Pigs. Animals.

[77] Bai Y., Zhou X., Zhao J., Wang Z., Ye H., Pi Y., Che D., Han D., Zhang S., Wang J. (2022). Sources of dietary fiber affect the SCFA production and absorption in the hindgut of growing pigs. Front. Nutr..

[78] Atasoy M., Eyice O., Schnürer A., Cetecioglu Z. (2019). Volatile fatty acids production via mixed culture fermentation: Revealing the link between pH, inoculum type and bacterial composition. Bioresour. Technol..

[79] Jørgensen H., Larsen T., Zhao X.-Q., Eggum B.O. (1997). The energy value of short-chain fatty acids infused into the caecum of pigs. Br. J. Nutr..

[80] Diao H., Jiao A.R., Yu B., Mao X.B., Chen D.W. (2019). Gastric infusion of short-chain fatty acids can improve intestinal barrier function in weaned piglets. Genes Nutr..

[81] Liu X.-F., Shao J.-H., Liao Y.-T., Wang L.-N., Jia Y., Dong P.-J., Liu Z.-Z., He D.-D., Li C., Zhang X. (2023). Regulation of short-chain fatty acids in the immune system. Front. Immunol..

[82] La A.L.T.Z., Feng Y., Hu D., Feng Y., Jin X., Liu D., Guo Y., Cheng G., Hu Y. (2023). Enzymatically prepared alginate oligosaccharides improve broiler chicken growth performance by modulating the gut microbiota and growth hormone signals. J. Anim. Sci. Biotechnol..

[83] Tsukahara T., Kishino E., Inoue R., Nakanishi N., Nakayama K., Ito T., Ushida K. (2013). Correlation between villous height and the disaccharidase activity in the small intestine of piglets from nursing to growing. Anim. Sci. J..

[84] Wang M., Yang C., Wang Q., Li J., Huang P., Li Y., Ding X., Yang H., Yin Y. (2020). The relationship between villous height and growth performance, small intestinal mucosal enzymes activities and nutrient transporters expression in weaned piglets. J. Anim. Physiol. Anim. Nutr..

[85] Pluske J.R. (2001). Morphological and functional changes in the small intestine of the newly-weaned pig. Gut Environment of Pigs.

[86] Pluske J.R., Hampson D.J., Williams I.H. (1997). Factors influencing the structure and function of the small intestine in the weaned pig: A review. Livest. Prod. Sci..

[87] Li Y., Huang T.-T., Carlson E.J., Melov S., Ursell P.C., Olson J.L., Noble L.J., Yoshimura M.P., Berger C., Chan P.H. (1995). Dilated cardiomyopathy and neonatal lethality in mutant mice lacking manganese superoxide dismutase. Nat. Genet..

[88] Velarde M.C., Flynn J.M., Day N.U., Melov S., Campisi J. (2012). Mitochondrial oxidative stress caused by Sod2 deficiency promotes cellular senescence and aging phenotypes in the skin. Aging.

[89] Günzel D., Yu A.S.L. (2013). Claudins and the Modulation of Tight Junction Permeability. Physiol. Rev..

[90] Elahi S., Buchanan R.M., Attah-Poku S., Townsend H.G.G., Babiuk L.A., Gerdts V. (2006). The Host Defense Peptide Beta-Defensin 1 Confers Protection against *Bordetella pertussis* in Newborn Piglets. Infect. Immun..

[91] Grondin J.A., Kwon Y.H., Far P.M., Haq S., Khan W.I. (2020). Mucins in Intestinal Mucosal Defense and Inflammation: Learning from Clinical and Experimental Studies. Front. Immunol..

[92] Menon B.B., Kaiser-Marko C., Spurr-Michaud S., Tisdale A.S., Gipson I.K. (2015). Suppression of Toll-like receptor-mediated innate immune responses at the ocular surface by the membrane-associated mucins MUC1 and MUC16. Mucosal Immunol..

[93] Layunta E., Buey B., Mesonero J.E., Latorre E. (2021). Crosstalk Between Intestinal Serotonergic System and Pattern Recognition Receptors on the Microbiota–Gut–Brain Axis. Front. Endocrinol..

[94] Liu Y.D., Yu L., Ying L., Balic J., Gao H., Deng N.T., West A., Yan F., Ji C.B., Gough D. (2019). Toll-like receptor 2 regulates metabolic reprogramming in gastric cancer *via* superoxide dismutase 2. Int. J. Cancer.

[95] Gibson D.L., Ma C., Rosenberger C.M., Bergstrom K.S.B., Valdez Y., Huang J.T., Khan M.A., Vallance B.A. (2008). Toll-like receptor 2 plays a critical role in maintaining mucosal integrity during Citrobacter rodentium-induced colitis. Cell. Microbiol..

[96] Arnaud E., Gardiner G.E., Chombart M., Doherty J.V.O., Sweeney T., Lawlor P.G. (2024). Effect of creep feeding pelleted starter diet, liquid milk replacer and a liquid mixture of starter diet and milk replacer to suckling pigs on their growth and medication usage. Transl. Anim. Sci..

[97] O’Doherty J.V., Kiernan D.P., Sweeney T., Wiseman J. (2024). Gastrointestinal development in pigs: Implications for nutrition and performance. Advances in Pig Nutrition.

